# Post-Transplantation Cyclophosphamide Uniquely Restrains Alloreactive CD4^+^ T-Cell Proliferation and Differentiation After Murine MHC-Haploidentical Hematopoietic Cell Transplantation

**DOI:** 10.3389/fimmu.2022.796349

**Published:** 2022-02-15

**Authors:** Ashley D. Hadjis, Natalia S. Nunes, Shanzay M. Khan, Rochelle E. Fletcher, Alessandra de Paula Pohl, David J. Venzon, Michael A. Eckhaus, Christopher G. Kanakry

**Affiliations:** ^1^ Experimental Transplantation and Immunotherapy Branch, Center for Cancer Research, National Cancer Institute, National Institutes of Health, Bethesda, MD, United States; ^2^ Biostatistics and Data Management Section, Office of the Clinical Director, Center for Cancer Research, National Cancer Institute, National Institutes of Health, Bethesda, MD, United States; ^3^ Division of Veterinary Resources, Office of Research Services, National Institutes of Health, Bethesda, MD, United States

**Keywords:** graft-versus-host disease (GVHD), haploidentical, alloreactive, bendamustine, methotrexate, regulatory (Treg) cell, post-transplantation cyclophosphamide (PTCy), allogeneic hematopoietic cell transplantation (HCT)

## Abstract

Post-transplantation cyclophosphamide (PTCy) reduces the incidence and severity of graft-versus-host disease (GVHD), thereby improving the safety and accessibility of allogeneic hematopoietic cell transplantation (HCT). We have shown that PTCy works by inducing functional impairment and suppression of alloreactive T cells. We also have identified that reduced proliferation of alloreactive CD4^+^ T cells at day +7 and preferential recovery of CD4^+^CD25^+^Foxp3^+^ regulatory T cells (T_regs_) at day +21 are potential biomarkers associated with optimal PTCy dosing and timing in our B6C3F1→B6D2F1 MHC-haploidentical murine HCT model. To understand whether the effects of PTCy are unique and also to understand better the biology of GVHD prevention by PTCy, here we tested the relative impact of cyclophosphamide compared with five other optimally dosed chemotherapeutics (methotrexate, bendamustine, paclitaxel, vincristine, and cytarabine) that vary in mechanisms of action and drug resistance. Only cyclophosphamide, methotrexate, and cytarabine were effective in preventing fatal GVHD, but cyclophosphamide was superior in ameliorating both clinical and histopathological GVHD. Flow cytometric analyses of blood and spleens revealed that these three chemotherapeutics were distinct in constraining conventional T-cell numerical recovery and facilitating preferential T_reg_ recovery at day +21. However, cyclophosphamide was unique in consistently reducing proliferation and expression of the activation marker CD25 by alloreactive CD4^+^Foxp3^-^ conventional T cells at day +7. Furthermore, cyclophosphamide restrained the differentiation of alloreactive CD4^+^Foxp3^-^ conventional T cells at both days +7 and +21, whereas methotrexate and cytarabine only restrained differentiation at day +7. No chemotherapeutic selectively eliminated alloreactive T cells. These data suggest that constrained alloreactive CD4^+^Foxp3^-^ conventional T-cell numerical recovery and associated preferential CD4^+^CD25^+^Foxp3^+^ T_reg_ reconstitution at day +21 may be potential biomarkers of effective GVHD prevention. Additionally, these results reveal that PTCy uniquely restrains alloreactive CD4^+^Foxp3^-^ conventional T-cell proliferation and differentiation, which may explain the superior effects of PTCy in preventing GVHD. Further study is needed to determine whether these findings also hold true in clinical HCT.

## Introduction

Allogeneic hematopoietic cell transplantation (HCT) is the only potentially curative therapy for many life-threatening hematologic diseases, but historically was not accessible to many patients for lack of a suitable human leukocyte antigen (HLA)-matched donor. HLA-haploidentical donors are available for nearly all patients, but early results of HLA-haploidentical HCT showed unacceptably high rates of graft failure, graft-versus-host disease (GVHD), and transplant-related mortality due to strong bi-directional alloreactivity (1). The administration of the chemotherapeutic cyclophosphamide on days +3 and +4 post-transplant (post-transplantation cyclophosphamide, PTCy) can greatly reduce the incidence and severity of acute and chronic graft-versus-host disease (GVHD) after HLA-haploidentical or HLA-partially mismatched unrelated donor HCT and consequently has been widely adopted (2). Despite very encouraging clinical outcomes, how PTCy works to prevent GVHD has not been well understood.

A better understanding of the immunological mechanisms by which PTCy works to prevent GVHD may allow for rational modifications of this platform in attempts to improve outcomes for patients. In murine HCT models, we have shown that PTCy works by inducing alloreactive T-cell functional impairment and subsequent suppression by CD4^+^Foxp3^+^ regulatory T cells (T_regs_) (3–5). We also have shown in our B6C3F1→B6D2F1 MHC-haploidentical murine HCT model that optimal dosing and timing of PTCy are associated with reduced proliferation of alloreactive CD4^+^Foxp3^-^ conventional T cells at day +7 and preferential recovery of CD4^+^CD25^+^Foxp3^+^ T_regs_ at day +21 (5, 6). We have proposed that together these T-cell endpoints may be potential biomarkers of effective GVHD control by PTCy (6).

The relative survival and recovery of T-cell subsets after optimally timed and dosed PTCy may be due to differential and dynamic expression of important drug resistance pathways, including aldehyde dehydrogenase (ALDH) and ATP-binding cassette (ABC) transporters (3, 4, 7). Human and mouse T_regs_ upregulate expression of ALDH, the main *in vivo* detoxifying pathway for cyclophosphamide (8), after alloantigen stimulation, contributing to resistance to cyclophosphamide-induced cell death in this specific context (3, 4, 9). These pathways also contribute to human CD8^+^ T-cell survival and recovery after cyclophosphamide (7). ALDH and ABC transporters are not only widely expressed throughout the hematopoietic system (10, 11), but also confer differential degrees of resistance to virtually all classes of chemotherapeutics (12–14). Each chemotherapeutic also may have additional specific mechanisms of resistance.

It is unclear whether the effects of cyclophosphamide (CY) given in the early post-transplant period are unique. In 1971, survival after murine MHC-haploidentical HCT was compared after treatment with cyclophosphamide, methotrexate, mercaptopurine, chlormethine, or cortisol, each administered on days +5, +8, +11, and +14 (15). Cyclophosphamide was the only drug found to be effective, while all other drugs had minimal impact (15). However, since 1971, not only are there more chemotherapeutics available, but we also have identified that PTCy is maximally effective in HCT when given between days +3 to +5 (6). Additionally, the relative effects of PTCy compared with other chemotherapeutics on T-cell subsets have not been examined. The only exception has been recent interest in post-transplantation bendamustine (BEN), which has been shown in pre-clinical studies to produce engraftment and GVHD prevention results similar to PTCy, while maintaining the graft-versus-leukemia effect (16–18); yet, phase I/II trials thus far have shown mixed results (17, 18).

To assess whether the biological effects of PTCy may be unique and provide further insights into our mechanistic understanding of the immunological mechanisms by which PTCy prevents GVHD, we investigated in our murine MHC-haploidentical HCT model (5) the relative efficacy of five other chemotherapeutics (methotrexate, bendamustine, paclitaxel, vincristine, and cytarabine). We specifically chose these drugs as they represent an array of mechanisms of action, metabolism, and drug resistance (**Table 1**) and include methotrexate, which has a long history of clinical use for GVHD prophylaxis, and bendamustine, which has recently been explored as an alternative to PTCy; topoisomerase inhibitors and other alkylators beyond bendamustine were intentionally excluded over theoretical concern for therapy-related myeloid neoplasms in any clinical application of these studies.

**Table 1:** Putative pathways of resistance to chemotherapeutics tested in this study as assessed by available literature (8, 12–14, 19–44).

Drug	Class	ALDH	ABC	Other Mechanisms of Resistance
**Cyclophosphamide (CY)**	Alkylating agent	+++	+	Inactivation *via* glutathione S-transferase
**Bendamustine (BEN)**	Alkylating agent + antimetabolite	NR	+	Only partial cross-resistance to other alkylating agents. Mechanisms of resistance understudied.
**Methotrexate (MTX)**	Antimetabolite (antifolate)	+	++	Reduced uptake *via* the human reduced folate carrier. Increased dihydrofolate reductase activity. Decreased polyglutamylation.
**Cytarabine (ARA-C)**	Antimetabolite (antinucleoside)	+	+	Reduced uptake *via* human equilibrative nucleoside transporter. Decreased activation *via* deoxycytidine kinase deficiency or increased expression of 5’ nucleotidases. Deactivation *via* increased expression of cytidine deaminase. Increased expression of DNA polymerase α.
**Paclitaxel (PTX)**	Antimicrotubular/taxane	+	++	Alterations to the tubulin/microtubule system.
**Vincristine (VCR)**	Antimicrotubular/vinca alkaloid	+	++	Alterations to the tubulin/microtubule system.

+, some evidence in literature suggesting involvement; ++, more numerous reports of involvement and/or likely a major mediator of resistance, but other pathways also may play important roles; +++ established involvement as a key mediator of resistance. NR, not reported.

## Materials and Methods

### Mice

B6C3F1/Crl (donor) and B6D2F1/Crl (recipient) female mice, 10-12 weeks old at the time of transplant, were obtained from the Charles River Laboratories. Mice were housed in specific-pathogen-free conditions at the NCI and were provided food and water *ad libitum*.

### HCT

Spleens, tibias, and femurs were aseptically collected from donor B6C3F1 mice and processed as previously described, including red blood cell lysis of splenocytes and T-cell depletion of bone marrow (5). Recipient B6D2F1 mice were irradiated to 10.5 Gy in a single fraction and 6-8 hours later received 10x10^6^ B6C3F1 T-cell-depleted bone marrow +/- 40x10^6^ red blood cell-depleted B6C3F1 splenocytes *via* tail vein injection. Recipient mice received levofloxacin-treated water from days 0 to +14. Survival was followed daily, and blinded assessments of weights and clinical scores using a standardized rubric (5) were performed every three days. Tissue specimen preparation and blinded histopathologic assessments were performed as previously described (5).

### Drug Preparation

On the day of administration, methotrexate (MTX, Intas Pharmaceuticals), paclitaxel (PTX, Athenex), vincristine (VCR, Hospira), and cytarabine (ARA-C, Hospira) were diluted with sterile PBS to appropriate concentrations, while bendamustine (BEN, TEVA Pharmaceutical Industries) was reconstituted using sterile water to 5 mg/ml per the manufacturer’s instructions and then further diluted to the appropriate concentrations with sterile PBS. Cyclophosphamide (CY, Baxter Oncology) was prepared as previously described (5) and diluted to a 1 mg/ml concentration with sterile PBS on the day of administration for injection. All drugs were diluted to concentrations allowing for administration of 300-500 µl per mouse and administered *via* intraperitoneal injection. Doses were based on the weight on the day of injection. Vehicle-treated mice received similar volumes of sterile PBS intraperitoneally.

### Flow Cytometry

Blood and spleens were collected and processed as previously described (5). Viable cell counts were performed with dual-fluorescent imaging with a Cellometer Auto 2000 Cell Viability Counter (Nexcelom). 2x10^6^ viable cells/sample were stained sequentially with LIVE/DEAD Fixable Aqua Dead Cell Stain Kit (Thermo Fisher), extracellular antibodies, fixation/permeabilization (eBioscience Foxp3/Transcription Factor Staining Kit), and intracellular antibodies. Single stains were used to generate compensations, and fluorescence-minus-one controls were prepared for CD25, Ki-67, and phospho-STAT5. Data were acquired on an LSRFortessa (BD Biosciences) and analyzed using FCS Express (De Novo Software). Cell subsets had to be at least 50 cells to allow for reliable further subsetting; subsets with denominators less than this threshold were excluded from further subsetting analyses.

Fluorochrome-conjugated monoclonal antibodies used for flow cytometry included BUV395 anti-CD3 (clone 145-2C11), BV786 anti-CD8a (clone 53-6.7), PE-CF594 anti-CD25 (clone PC61), AF700 anti-CD44 (clone IM7), BUV737 anti-CD62L (clone MEL-14), PE anti-H2k^k^ (clone 36-7-5), and BV711 anti-H2k^k^ (clone AF3-12.1) from BD Biosciences; PE-Cy5 anti-CD8 (clone 53-6.7), PE-Cy7 anti-H2k^d^ (clone SF1-1.1), and BV605 anti-Ki67 (clone 16A8) from BioLegend; and APC-eFluor780 anti-CD4 (clone GK1.5), PerCP-eFluor710 anti-Vβ6 (clone RR4-7), eFluor450 anti-Foxp3 (clone FJK-16s), and PE anti-phospho-STAT5 (Tyr694) (clone SRBCZX) from Invitrogen.

### Statistics

Survival distributions were compared using the exact log-rank test. Weight and clinical score area-under-the-curve (AUC) comparisons were performed using Wilcoxon’s rank sum test and were restricted to intervals in which ≥70% of vehicle-treated mice survived. Weight and clinical score data are shown as the mean +/- SEM. Due to strong serial correlations, weight and clinical score SEMs were not corrected for multiple measurements. Cell counts and median fluorescence intensities were natural logarithmically transformed and cell subset percentages were transformed using an arcsine transformation prior to one-way ANOVA. ANOVA results were followed with the Holm-Sidak *post hoc* correction for the multiple comparisons to the control group. Non-transformed data are displayed and are shown as box-and-whisker plots for ease of understanding, but transformed data were used for statistical testing. SAS/STAT software (SAS Institute Inc.), version 14.3, was used for analyses of survival, weight, and clinical score data. GraphPad Prism (GraphPad Software), version 8.4.3 was used for all other statistical analysis. *P* values <0.05 were considered statistically significant in an exploratory mode of analysis of repeated measurements of correlated immunologic outcomes.

### Study Approval

Mice were treated under a protocol approved by the NCI Animal Care and Use Committee in accordance with the NIH Guide for the Care and Use of Laboratory Animals.

## Results

### MTX 5 mg/kg/day, BEN 10 mg/kg/day, PTX 1 mg/kg/day, VCR 0.05 mg/kg/day, and ARA-C 25 mg/kg/day Are Optimal Doses of Each Drug When Given on Days +3 and +4 in the B6C3F1→B6D2F1 MHC-Haploidentical HCT Model

**Figure 1:**
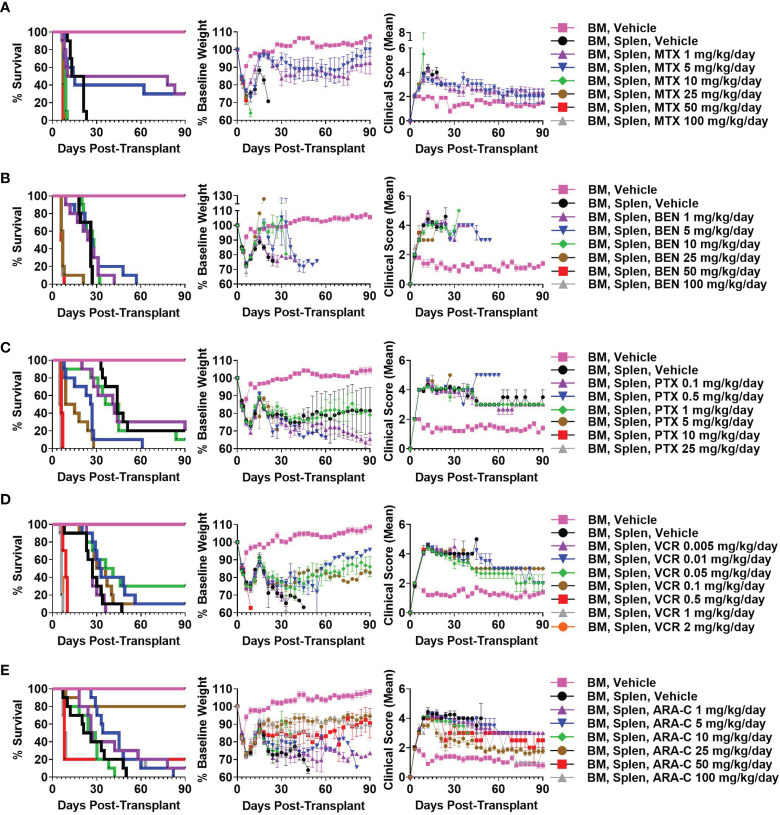
Methotrexate (MTX) 5 mg/kg/day, bendamustine (BEN) 10 mg/kg/day, paclitaxel (PTX) 1 mg/kg/day, vincristine (VCR) 0.05 mg/kg/day, and cytarabine (ARA-C) 25 mg/kg/day are optimal doses of each drug when given on days +3/+4 in the B6C3F1 → B6D2F1 MHC-haploidentical hematopoietic cell transplantation (HCT) model. On day 0, recipient 10-12-week-old female B6D2F1 mice were irradiated to 10.5 Gy in a single fraction and transplanted 6-8 hours later *via* intravenous injection with 10 x 10^6^ T-cell-depleted bone marrow (BM) cells +/- 40 x 10^6^ red-blood-cell-depleted splenocytes (Splen) from 10-12-week-old female B6C3F1 donors. Phosphate buffered saline (PBS) vehicle or the chemotherapeutic of interest was administered intraperitoneally on days +3 and +4. Although different chemotherapeutics had varying efficacy in mitigating fatal or severe graft-versus-host disease (GVHD), **(A)** MTX 5 mg/kg/day, **(B)** BEN 10 mg/kg/day, **(C)** PTX 1 mg/kg/day, **(D)** VCR 0.05 mg/kg/day, and **(E)** ARA-C 25 mg/kg/day were determined as optimal doses for each drug due to superior survival, weights, and/or clinical scores. Combined results from two independent experiments of n = 5 mice/group/experiment are shown.

We have previously shown that PTCy is maximally effective in our B6C3F1→B6D2F1 MHC-haploidentical HCT model when given between days +3 to +5 with dosing on days +3 and +4 being among the best dosing schedules and also is what is used clinically (2, 6). Therefore, we sought to identify the best dose of each alternative chemotherapeutic (MTX, BEN, PTX, VCR, ARA-C) when given on days +3 and +4 in this HCT model; we explored a wide range of doses, spanning what were thought to be below effective doses to near-lethal ranges based on prior studies (15, 16, 19, 45–56). MTX 5 mg/kg/day, BEN 10 mg/kg/day, PTX 1 mg/kg/day, VCR 0.05 mg/kg/day, and ARA-C 25 mg/kg/day were determined as best for each drug, although some had minimal to no efficacy in preventing fatal GVHD (**Figure 1** and **Supplementary Figure 1**).

### CY 25 mg/kg/day Is Superior to All Other Chemotherapeutics in Preventing Severe GVHD

**Figure 2:**
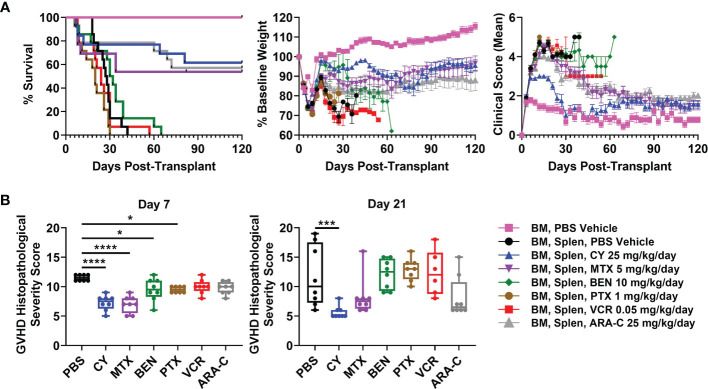
Post-transplantation cyclophosphamide (PTCy), MTX, and ARA-C all are partially effective in mitigating severe GVHD with PTCy having the most efficacy. Mice were transplanted as in **Figure 1** and were given either PBS or the previously determined optimal dose of one of the tested chemotherapeutics. **(A)** CY (HR 0.12, p = 0.0008), MTX (HR 0.28, p = 0.012), and ARA-C (HR 0.11, p = 0.0004) all significantly prolonged survival compared with vehicle-treated mice, whereas PTX led to more rapid mortality (HR 2.42, p = 0.038). MTX and ARA-C had similar survival compared with CY, but BEN (HR 6.86, p = 0.0008), PTX (HR 8.41, p = 0.0005), and VCR (HR 5.86, p = 0.0012) had worse survival. However, of the three partially effective chemotherapeutics, only CY-treated mice had significantly higher weights (p = 0.015) compared with vehicle-treated mice (p > 0.10 for MTX and ARA-C). Conversely, VCR-treated mice had significantly lower weights (p = 0.011) and PTX trended towards lower weights (p = 0.075) compared with vehicle-treated mice. CY-treated mice had higher weights compared with PTX-treated (p = 0.0068), VCR-treated (p = 0.0011), and ARA-C-treated (p = 0.02) mice and also had marginally higher weights compared with MTX-treated mice (p = 0.052). CY (p < 0.0001), MTX (p = 0.03), and ARA-C (p < 0.0001) led to better clinical scores than vehicle-treated mice, and CY was significantly better than all other treatment groups including both MTX and ARA-C (p < 0.0001 for each). Statistical comparisons for clinical scores and weights are for the area-under-the-curve (AUC) calculations over the period of time in which ≥70% of vehicle-treated mice were alive. **(B)** Mice were taken for histopathology of GVHD target organs on day +7 or +21. Several chemotherapeutics (CY, MTX, BEN, PTX) significantly reduced histopathological GVHD at day +7, but only CY continued to significantly reduce histopathological GVHD at day +21. *p < 0.05, ***p < 0.001, ****p < 0.0001 on one-way ANOVA followed by the Holm-Sidak *post hoc* test using the vehicle-treated group as the control. Only significant results are shown; all other comparisons between treatment groups and the vehicle group are non-significant. Combined results from two independent experiments are shown; n = 5/group/experiment for the weights and clinical score assessments in A except TCD BM + PBS (n = 9 total), and n = 4/group/experiment for all groups in B except VCR at day +21 (n = 6 total) due to excess early deaths. Extra mice set up in B for both experiments were also followed and included in the survival graphs [n = 4 independent experiments with total n=14/group except CY (n = 13), MTX (n = 13), and TCD BM + PBS vehicle (n = 9)].

We next compared the optimal doses of these other chemotherapeutics with the previously established optimal dose of CY (25 mg/kg/day) in this MHC-haploidentical HCT model (5, 6), with all drugs being administered as daily doses on days +3 and +4. CY, MTX, and ARA-C all were partially effective in preventing fatal GVHD (**Figure 2A**). BEN slightly delayed survival in a subset of mice, while VCR and PTX were completely ineffective in preventing fatal GVHD (**Figure 2A**). In fact, PTX led to more rapid mortality (**Figure 2A**).

CY was superior to all other chemotherapeutics in ameliorating clinical GVHD (**Figure 2A**). CY-treated mice had significantly improved body weights and clinical scores compared with vehicle-treated mice as well as with MTX- and ARA-C-treated mice (**Figure 2A**). MTX and ARA-C had similar weights but significantly better clinical scores than vehicle-treated mice. BEN, VCR, and PTX did not significantly improve clinical scores compared with vehicle-treated mice (**Figure 2A**), while VCR had significantly lower body weights and PTX trended towards lower body weights compared with vehicle-treated mice (**Figure 2A**). Higher mean body weight in BEN-treated mice was a result of substantial weight gain from ascites, rather than clinical benefit from the chemotherapeutic (**Figure 2A**). Autopsy suggested that these mice were developing ascites secondary to GVHD-induced liver failure and protein-losing enteropathy, also evident in high average liver GVHD scores at day +21 (**Supplementary Figure 2**).

Histopathological scoring at day +7 revealed that mice receiving CY, MTX, BEN, and PTX all had significantly reduced total histopathological GVHD severity scores compared with vehicle-treated mice (**Figure 2B** and **Supplementary Table 1**). However, by day +21, only CY significantly reduced histopathological GVHD compared with vehicle-treated mice (**Figure 2B** and **Supplementary Table 2**). MTX and ARA-C both had lower median histopathological GVHD severity scores compared with vehicle-treated mice at day +21, but the lack of statistical significance was due to wider variability of scores between mice in those groups (**Figure 2B**).

### The Partially Effective Chemotherapeutics (CY, MTX, and ARA-C) All Constrain T-Cell Recovery at Day +21

**Figure 3:**
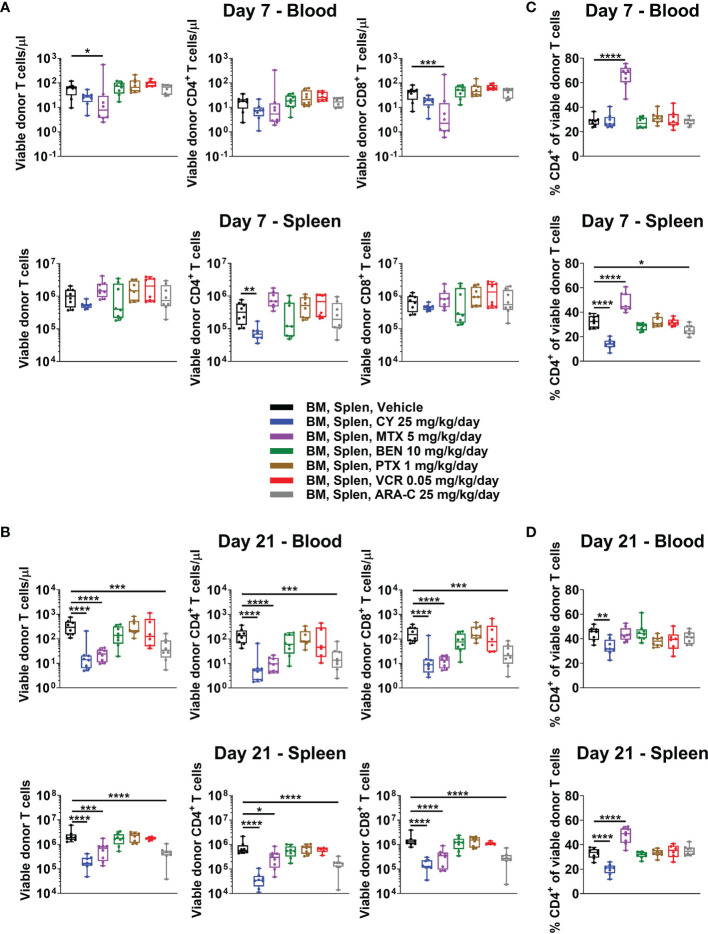
All partially effective chemotherapeutics constrain T-cell recovery at day +21, but MTX and CY differentially affect the balance of CD4^+^ versus CD8^+^ T cells, distinct from all other chemotherapeutics. Mice were transplanted as in **Figure 1** and received intraperitoneal injections on days +3 and +4 with either PBS vehicle or the optimal dose of one of the chemotherapeutics of interest. At day +7 or +21, mice were euthanized, and their blood and spleens were assessed by flow cytometry. **(A)** Total numbers of CD3^+^ T cells and CD4^+^ and CD8^+^ T-cell subsets were not significantly reduced in BEN-, PTX-, VCR-, or ARA-C-treated mice at day +7. However, MTX significantly reduced total number of CD3^+^ and CD8^+^ T cells in the blood, while CY significantly reduced total numbers of CD4^+^ T cells in spleens at day +7. **(B)** At day +21, CY, MTX, and ARA-C all constrained recovery of CD4^+^ and CD8^+^ T-cell subsets. **(C, D)** MTX and CY had opposite effects at both **(C)** day +7 and **(D)** day +21 on the balance of CD4^+^ and CD8^+^ T cells, divergent from effects seen in vehicle-treated mice and mice treated with other chemotherapeutics. Combined results from two independent experiments are shown with n = 4/group/experiment except for VCR at day +21 (n = 6 total) due to excess early deaths in one experiment prior to day +21. *p < 0.05, **p < 0.01, ***p < 0.001, ****p < 0.0001 on one-way ANOVA followed by the Holm-Sidak *post hoc* test using the vehicle-treated group as the control. Only significant results are shown; all other comparisons between treatment groups and the vehicle group are non-significant.

Some have contended that PTCy prevents GVHD *via in vivo* T-cell depletion, but our recent work showed that day +7 total numbers of T cells in blood, spleens, peripheral lymph nodes, and liver after CY 25 mg/kg/day, the optimal dose in this model, were similar to or in the same log range as vehicle-treated mice (5). Similar to these findings, total T-cell numbers in blood and spleen from CY and most other tested chemotherapeutics were not significantly reduced at day +7 when compared with vehicle-treated mice (**Figure 3A**). The only exception was that MTX reduced total T-cell concentrations in blood at day +7, which was attributable to a decrease in total CD8^+^ T-cell concentrations at that timepoint (**Figure 3A**). CY did not significantly reduce total T-cell numbers in either blood or spleen, but did result in a significant reduction of CD4^+^ T cells in the spleen at day +7 (**Figure 3A**). By contrast, CY, MTX, and ARA-C all significantly reduced total T-cell, including CD4^+^ and CD8^+^ T-cell subset, numbers at day +21, whereas the ineffective chemotherapeutics had total T-cell numbers similar to vehicle-treated mice (**Figure 3B**). This suggests that effective GVHD control may be associated with constrained T-cell recovery at day +21.

### The CD4^+^/CD8^+^ T-Cell Ratio Is Not Affected by Chemotherapeutics Other Than MTX, Which Increases It, and CY, Which Decreases It

MTX administration facilitated a markedly distinct recovery of CD4^+^ versus CD8^+^ T cells compared with all other treatment groups and opposite that of CY-treated mice (**Figures 3C, D** and **Supplementary Figure 3**). At day +7, MTX demonstrated higher percentages of CD4^+^ T cells in both blood and spleen compared with vehicle-treated mice, while CY-treated mice had similar percentages of CD4^+^ T cells in the blood and reduced percentages in the spleen (**Figure 3C**). At day +21, splenic CD4^+^ T-cell percentages remained high in MTX-treated mice, while CY again reduced percentages in both blood and spleens (**Figure 3D**). The ineffective chemotherapeutics all had similar percentages of CD4^+^ T cells compared with vehicle-treated mice across tissues at both days +7 and +21 (**Figures 3C, D**). This unique, contrasting T-cell recovery after MTX compared with that observed after PTCy is especially interesting considering that MTX was found to be effective in this model (**Figure 2**), albeit less so than PTCy, suggesting that MTX and PTCy may have distinct mechanisms of GVHD prevention.

### Unlike CY, MTX and ARA-C Do Not Control CD25 Expression by CD4^+^Foxp3^-^ Conventional T Cells at Day +7

**Figure 4:**
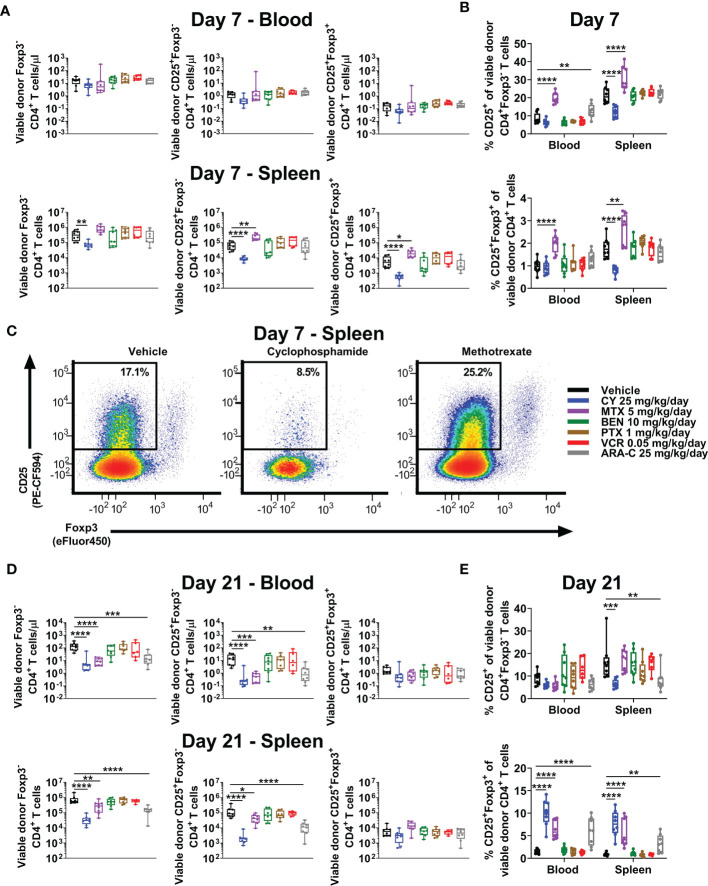
MTX and ARA-C do not control activated conventional CD4^+^ T cells at day +7, but at day +21 CY, MTX, and ARA-C (all partially effective chemotherapeutics) constrain conventional CD4^+^ T-cell recovery and facilitate preferential CD4^+^CD25^+^Foxp3^+^ regulatory T-cell recovery. Mice were transplanted, treated with PBS or a chemotherapeutic on days +3 and +4, and euthanized for flow cytometric assessment at day +7 or +21 as in **Figure 3**. **(A)** At day +7, total numbers of CD4^+^ T-cell subsets in the blood were not significantly different across treatment groups, but CY significantly reduced and MTX significantly increased total numbers of CD4^+^ T-cell subsets in the spleen. **(B)** Although MTX increased percentages of CD25^+^Foxp3^+^ regulatory T cells (T_regs_) at day +7 in both the blood and spleen, MTX also significantly increased percentages of conventional (Foxp3^-^) CD4^+^ T cells with an activated (CD25^+^) phenotype. This was distinct from CY, which reduced percentages of both. All other chemotherapeutics did not significantly alter these percentages in comparison with vehicle-treated mice except for ARA-C, which increased percentages of activated (CD25^+^) conventional CD4^+^ T cells only in the blood. **(C)** Representative flow cytometric plots are shown of CD4^+^ T cells gated on CD25 versus Foxp3 expression, showing percentages of CD25^+^Foxp3^-^ CD4^+^ T cells at day +7 that were decreased after CY but increased after MTX. CD25-positivity was gated based on the use of a fluorescence-minus-one (FMO) control. **(D)** At day +21, total numbers of Foxp3^-^ and CD25^+^Foxp3^-^ conventional CD4^+^ T cells were decreased in mice treated with the partially effective chemotherapeutics (CY, MTX, and ARA-C), whereas total numbers of CD4^+^CD25^+^Foxp3^+^ T_regs_ were similar across treatment groups. **(E)** Due to this balance, CY, MTX, and ARA-C all were associated with increased percentages of CD4^+^CD25^+^Foxp3^+^ T_regs_ at day +21, while CY and ARA-C also reduced the percentages of activated (CD25^+^) conventional CD4^+^ T cells. Combined results from two independent experiments are shown with n = 4/group/experiment for **(A–E)** except for VCR (n = 6 total) in **(D, E)** due to excess early deaths. *p < 0.05, **p < 0.01, ***p < 0.001, ****p < 0.0001 on one-way ANOVA followed by the Holm-Sidak *post hoc* test using the vehicle-treated group as the control. Only significant results are shown; all other comparisons between treatment groups and the vehicle group are non-significant.

Consistent with increased percentages of CD4^+^ T cells in MTX-treated mice (**Figure 3C**), total numbers of CD4^+^Foxp3^-^ conventional T cells were similar to slightly higher in MTX-treated compared with vehicle-treated mice at day +7 (**Figure 4A**). Furthermore, at day +7, much higher percentages of CD4^+^Foxp3^-^ conventional T cells appeared to have an activated phenotype (CD25^+^Foxp3^-^) in MTX-treated mice and, to a lesser extent, ARA-C-treated mice, whereas this percentage was reduced in CY-treated mice (**Figures 4B, C**). Interestingly, this difference in CD25 expression of CD4^+^Foxp3^-^ conventional T cells did not equate to differences in STAT5 phosphorylation, which was not decreased in CY-treated mice (**Supplementary Figure 4**). Corresponding to the higher CD25 expression within CD4^+^Foxp3^-^ conventional T cells after MTX and ARA-C at day +7, MTX- and ARA-C-treated mice had worse weights and clinical scores than CY-treated mice (**Figure 2A**). This clinical effect was seen despite increased percentages of CD4^+^CD25^+^Foxp3^+^ T_regs_ at day +7 in MTX-treated mice, whereas these percentages were reduced in the spleens of CY-treated mice at this timepoint (**Figure 4B**).

### All Partially Effective Chemotherapeutics Facilitate Preferential T_reg_ Recovery at Day +21

We have shown that T_regs_ are necessary for GVHD prevention by PTCy and that this role is increasingly important as time progresses post-transplant in suppressing surviving alloreactive T cells (3–5). PTCy also allows preferential recovery of T_regs_ in mice and patients (3–6); indeed, increased percentages of T_regs_ at day +21 are associated in our MHC-haploidentical HCT model with more effective dosing schedules of PTCy (5, 6). Consistent with our previous work, CY facilitated increased percentages of T_regs_ at day +21 in both blood and spleens (**Figures 4D, E**), an effect that also was seen with the two other partially effective chemotherapeutics, MTX and ARA-C (**Figures 4D, E**). Conversely, the ineffective drugs, BEN, PTX, and VCR, were not associated with increased percentages of T_regs_ at either timepoint (**Figures 4B, E**). These data further support preferential recovery of T_regs_ at day +21 as a potential biomarker of successful GVHD prevention, as this T-cell endpoint has been consistent between all effective chemotherapeutics here and also for maximally effective dosing schedules of PTCy (5, 6).

### Alloreactive T Cells Persist After All Chemotherapeutics, but Alloantigen-Specific T_regs_ Are Increased at Day +7 After CY and MTX

**Figure 5:**
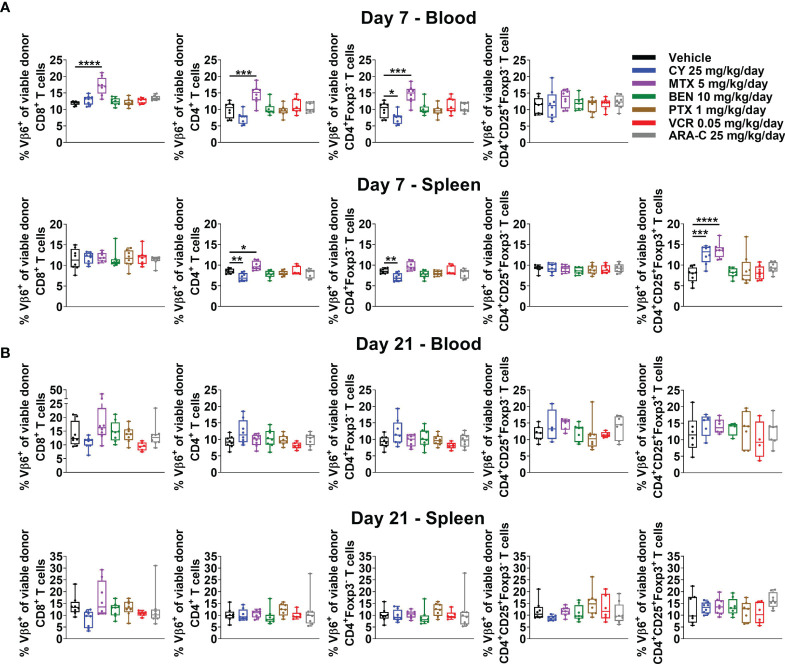
Alloreactive T cells persist after administration of all chemotherapeutics, but alloantigen-specific T_regs_ are increased at day +7 after CY and MTX. Mice were transplanted, treated with PBS or a chemotherapeutic on days +3 and +4, and euthanized for flow cytometric assessment at day +7 or +21 as in **Figure 3**. Alloreactive Vβ6^+^ T cells were not eliminated by any chemotherapeutic at either **(A)** day +7 or **(B)** day +21. **(A)** In fact, at day +7, percentages of CD8^+^, CD4^+^, and CD4^+^Foxp3^-^ T cells that were Vβ6^+^ were increased in MTX-treated mice. The percentages of CD4^+^Foxp3^-^ T cells that were Vβ6^+^ were slightly reduced after CY although other T-cell subsets, including percentages of CD4^+^CD25^+^Foxp3^-^ that were Vβ6^+^, were not affected. Interestingly, alloantigen-specific CD4^+^CD25^+^Foxp3^+^ cells were increased in CY- and MTX-treated mice at day +7 in the spleen; percentages in the blood were not included due to low total numbers of CD4^+^CD25^+^Foxp3^+^ cells at day +7 across treatment groups that did not permit reliable determination of further subsetting. **(B)** At day +21, there were no significant differences in percentages of Vβ6^+^ T cells across T-cell subsets or treatment groups. Combined results from two independent experiments are shown with n = 4/group/experiment except VCR (n = 6 total) in B due to excess early deaths. *p < 0.05, **p < 0.01, ***p < 0.001, ****p < 0.0001 on one-way ANOVA followed by the Holm-Sidak *post hoc* test using the vehicle-treated group as the control. Only significant results are shown; all other comparisons between treatment groups and the vehicle group are non-significant.

Previously PTCy was thought to work *via* selective elimination of alloreactive T cells, since these cells would be proliferating rapidly in the early post-transplant setting. However, our recently published work showed that PTCy does not selectively eliminate alloreactive T cells (5, 6). Indeed, neither after PTCy, nor after any of the other chemotherapeutics tested, were alloreactive T cells eliminated (**Figures 5A, B** and **Supplementary Figure 5**). At day +7, the percentages of CD4^+^Foxp3**^-^** T cells that were Vβ6^+^ were slightly reduced after PTCy (**Figure 5A**). By contrast, these percentages, and those of CD8^+^ T cells that were Vβ6^+^ actually were increased in the blood after MTX (**Figure 5A**). Interestingly, both PTCy and MTX were associated with increased percentages of alloantigen-specific (Vβ6^+^) T_regs_ at day +7 (**Figure 5A**). At day +21, across all T-cell subsets and treatment groups, there were no significant differences in percentages of alloreactive T cells compared with vehicle-treated mice (**Figure 5B**).

### CY Is the Only Effective Chemotherapeutic That Consistently Reduces Alloreactive CD4^+^ T-Cell Proliferation at Day +7

**Figure 6:**
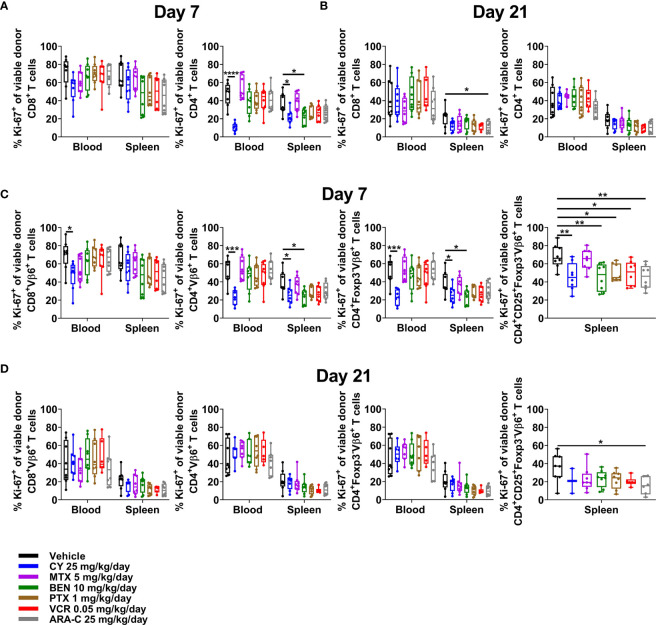
Only CY consistently reduces alloreactive CD4^+^ T-cell proliferation at day +7. Mice were transplanted, treated with PBS or a chemotherapeutic on days +3 and +4, and euthanized for flow cytometric assessment at day +7 or +21 as in **Figure 3**. **(A)** In both the blood and spleens at day +7, CY reduced proliferation (Ki-67^+^) of CD4^+^ T cells. **(B)** At day +21, proliferation was generally similar across all groups. **(C)** CY also reduced proliferation of alloreactive (Vβ6^+^) conventional CD4^+^ T cells at day +7. Interestingly, BEN, PTX, VCR, and ARA-C significantly reduced percentages of proliferating alloreactive CD4^+^CD25^+^Foxp3^-^ T cells at day +7, but did not affect proliferation of other alloreactive CD4^+^ T cells. **(D)** At day +21, proliferation of alloreactive subsets again was generally similar across all groups. Percentages of proliferating alloreactive CD4^+^CD25^+^Foxp3^-^ T cells in the blood are not shown in **(C, D)** due to low total numbers of CD4^+^CD25^+^Foxp3^-^Vβ6^+^ cells across treatment groups that did not permit reliable determination of further subsetting. Combined results from two independent experiments are shown with n = 4/group/experiment for **(A–D)** except for VCR (n = 6 total) in **(B, D)** due to excess early deaths. *p < 0.05, **p < 0.01, ***p < 0.001, ****p < 0.0001 on one-way ANOVA followed by the Holm-Sidak *post hoc* test using the vehicle-treated group as the control. Only significant results are shown; all other comparisons between treatment groups and the vehicle group are non-significant.

Although PTCy does not selectively eliminate alloreactive T cells, effective GVHD control by PTCy is associated with a decrease in percentages of CD4^+^, including alloreactive CD4^+^ T cells, that are proliferating at day +7 (5, 6). Consistent with previous results, CY reduced percentages of proliferating CD4^+^ T cells, including alloreactive (Vβ6^+^) conventional T-cell subsets, in both blood and spleens (**Figures 6A, C**). Conversely, neither MTX nor ARA-C significantly reduced proliferation of alloreactive T cells at day +7, except within CD4^+^CD25^+^Foxp3^-^Vβ6^+^ T cells after ARA-C (**Figure 6C**). By contrast, proliferation of alloreactive T cells had normalized and was similar across treatment groups and T-cell subsets at day +21 (**Figures 6B, D**).

### All Partially Effective Chemotherapeutics Restrain Alloreactive CD4^+^ Conventional T-Cell Differentiation at Day +7, but CY Has the Greatest Effect and Is the Only Drug That Maintains This Effect at Day +21

**Figure 7:**
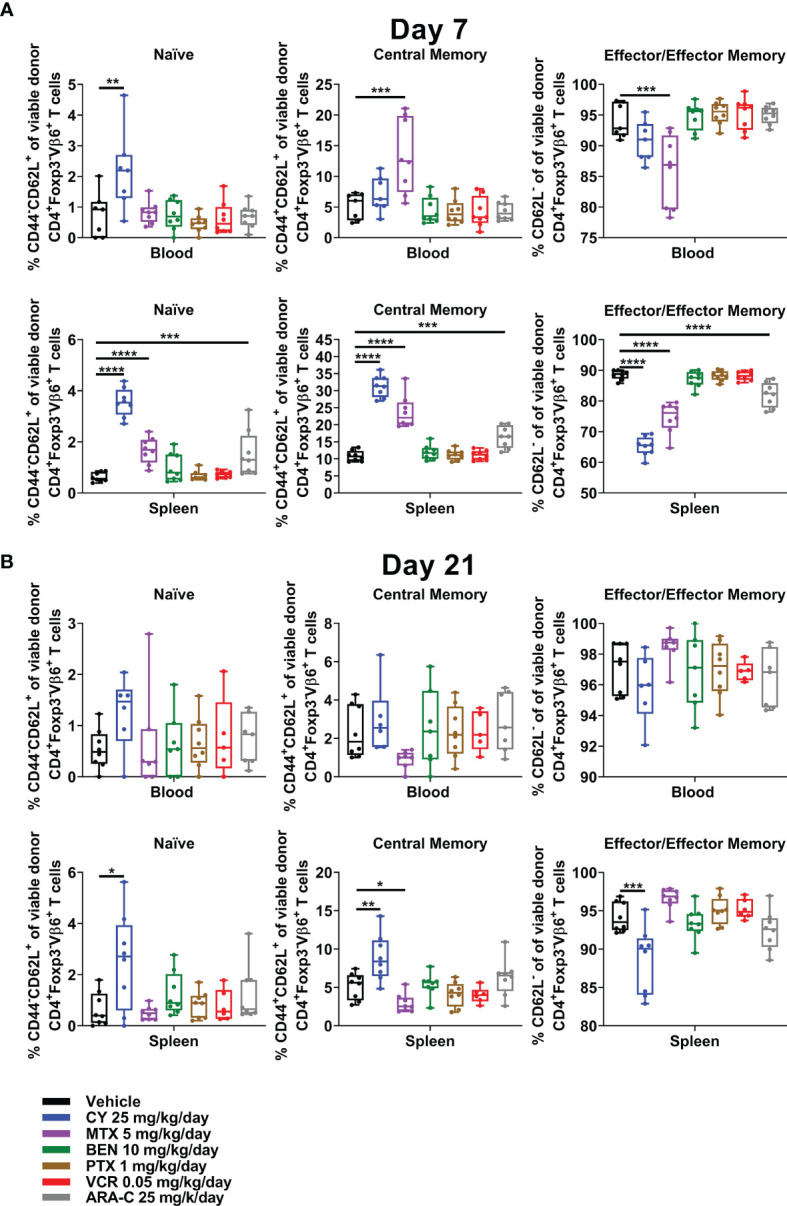
CY uniquely restrains T-cell differentiation at both early and later timepoints. Mice were transplanted, treated with PBS or a chemotherapeutic on days +3 and +4, and euthanized for flow cytometric assessment at day +7 or +21 as in **Figure 3**. **(A)** At day +7, CY decreased percentages of CD4^+^Foxp3^-^Vβ6^+^ T cells that were phenotypically effector/effector memory (CD62L^-^). Consequently, percentages of naïve (CD44^-^CD62L^+^) and central memory (CD44^+^CD62L^+^) CD4^+^Foxp3^-^Vβ6^+^ T cells were increased by CY at day +7. This same effect was achieved to a lesser extent after both MTX and ARA-C. **(B)** This restrained differentiation was persistent after CY at day +21 but was completely lost after MTX, wherein differentiation seemed to be overall accelerated. Combined results from two independent experiments are shown with n = 4/group/experiment except for VCR (n = 6 total) in **(B)** due to excess early deaths. *p < 0.05, **p < 0.01, ***p < 0.001, ****p < 0.0001 on one-way ANOVA followed by the Holm-Sidak *post hoc* test using the vehicle-treated group as the control. Only significant results are shown; all other comparisons between treatment groups and the vehicle group are non-significant.

CY, MTX, and ARA-C, the three chemotherapeutics that were partially effective in ameliorating GVHD, all were distinct in restraining alloreactive CD4^+^Foxp3^-^ T-cell differentiation at day +7, with less effector/effector memory and more naïve/central memory phenotypes (**Figure 7A**). This effect was most pronounced for CY and even more evident when looking at all CD4^+^Foxp3^-^ T cells (**Supplementary Figure 6**), which also would include alloreactive T cells beyond those that are Vβ6^+^. Furthermore, the restraint of alloreactive CD4^+^ T-cell differentiation continued after CY at day +21, whereas MTX-treated mice actually had slightly more highly differentiated alloreactive CD4^+^ T cells at that time point (**Figure 7B** and **Supplementary Figure 6**).

## Discussion

Using our previously described MHC-haploidentical murine HCT model (5), we tested the relative efficacy of five other chemotherapeutics (MTX, BEN, PTX, VCR, and ARA-C) in comparison with cyclophosphamide when given as GVHD prophylaxis early post-transplant. We demonstrated that PTCy not only was superior to all other tested chemotherapeutics in ameliorating severe GVHD clinically and histopathologically, but also showed that some of the effects of PTCy on T-cell subsets appear unique. Similar to PTCy, the other partially effective chemotherapeutics, MTX and ARA-C, did constrain conventional T-cell numerical recovery at day +21 and facilitated preferential recovery of T_regs_ at day +21. But, unlike PTCy, MTX and ARA-C did not reduce percentages of alloreactive CD4^+^ conventional T cells that were activated (as measured by CD25 expression) or proliferating at day +7 and only restrained alloreactive CD4^+^ conventional T-cell differentiation at day +7 (not day +21); indeed, MTX was associated with a more differentiated phenotype at day +21 compared with even vehicle-treated mice. These findings provide further support for reduced alloreactive CD4^+^ conventional T-cell proliferation at day +7 and preferential T_reg_ recovery at day +21 as potential biomarkers for effective GVHD control by PTCy as identified in our previous publications on optimal dosing and timing of PTCy (5, 6). Additionally, we propose restrained alloreactive CD4^+^ conventional T-cell activation and differentiation as possible additional elements that may further explain PTCy’s superior efficacy in preventing GVHD. The discrepancy between activation markers of CD25 and phospho-STAT5 positivity within CD4^+^ conventional T cells in PTCy-treated mice may suggest that activation is incomplete or impaired after PTCy, and we are working to better understand this phenomenon in the laboratory.

It is difficult to determine if PTCy causes preferential deletion of differentiated effector/effector memory alloreactive CD4^+^ conventional T cells or has a direct effect on alloreactive CD4^+^ conventional T cells that prevents their subsequent differentiation. Although this distinction is impossible to definitively tease out with this model system, the continued increased percentages of naïve/central memory cells and decreased percentages of effector/effector memory cells at day +21 seen in PTCy-treated mice would support the latter possibility. We are continuing to study in the laboratory the nature of the alloreactive T-cell dysfunction induced by PTCy that may contribute to GVHD prevention and restrained T-cell proliferation and differentiation.

PTCy previously was thought to work *via* the selective elimination of alloreactive T cells, as these cells would be rapidly proliferating in the early post-transplant time period. However, this hypothesis was extrapolated from data in MHC-matched murine skin-allografting models that were extremely contextual and had questionable relevance to HCT (5, 57–60). Our recently published work disproved this hypothesis in HCT, showing that alloreactive T cells persisted after PTCy administration even at high, intolerable PTCy doses (5). Both in our current and prior studies (5, 6), we did find a small transient reduction in the percentages of alloreactive CD4^+^ conventional T cells after PTCy in some organs, and here we also found that CD25-expressing activated alloreactive CD4^+^ conventional T cells were reduced. We cannot exclude that PTCy in some organs may preferentially diminish alloreactive T cells, particularly activated and proliferating alloreactive T cells, but any such effect is minor, incomplete, and short-lived. Alternatively, this effect could be explained by relative restraint of activation, proliferation, and expansion of surviving alloreactive T cells rather than selective killing, consistent with our findings of similar pSTAT5 signaling but reduced CD25 expression in PTCy-treated mice. Even so, all our prior and current data clearly show that alloreactive T cells are not selectively eliminated by PTCy (5, 61).

In retrospect, the lack of selective alloreactive T-cell elimination should not be surprising since cyclophosphamide, as an alkylator, is a non-cell-cycle-specific chemotherapeutic. Thus, it appears that PTCy may be killing a substantial percentage of T cells in a dose-dependent manner, but this effect is broad and not selective for alloreactive T cells (5, 61). Interestingly, none of the chemotherapeutics tested in these experiments selectively eliminated alloreactive T cells, despite the use of some cell-cycle-specific chemotherapeutic agents; by contrast, the percentages of alloreactive T cells were even higher after MTX at day +7. Even though none of these other chemotherapeutics greatly affected either the relative percentages of alloreactive T cells or global T-cell proliferation, all drugs except MTX did slightly reduce the proliferation of activated (CD25^+^) alloreactive CD4^+^ conventional T cells at day +7. Overall though, the decline was modest and not associated with substantial clinical or histopathologic reduction in GVHD severity for most drugs.

Moreover, no chemotherapeutic induced pan T-cell depletion at day +7 compared with vehicle-treated mice, which may in part be attributable to intermediate rather than maximally tolerated doses being tested as intermediate doses were the most optimal for survival for each chemotherapeutic. Even so, we have previously shown that very high doses of PTCy, which did greatly reduce T-cell counts at day +7, resulted in preferential survival of alloreactive T cells at day +7 and actually led at day +21 to a tremendous rebound in alloreactive T-cell counts, consequent blunting of regulatory T-cell recovery, and worse GVHD in our model compared with intermediate dosing (5). Indeed, early results from a PTCy dose de-escalation clinical study at our institution suggest that intermediate-dose PTCy maintains excellent protection against acute GVHD (62). Whether such results also are true for other chemotherapeutics is unknown, but is important to understand, particularly given the recent attempts to implement post-transplantation bendamustine clinically (17, 18).

A limitation of this study is that all the alternative chemotherapeutics tested were administered on days +3/+4 to best compare against PTCy each chemotherapeutic’s effects on GVHD and immune subsets; PTCy is administered in this manner in clinical practice and administration on days +3/+4 is among the most effective PTCy dosing schedules in our MHC-haploidentical murine HCT model (6). We have hypothesized that PTCy administration on days +3/+4 is particularly effective because during this critical window specific T-cell subsets are metabolically primed, based on differential dynamic expression of ALDH and ABC transporter activity, for differential sensitivity to cyclophosphamide (3–7, 9). In previous experiments using our MHC-haploidentical murine HCT model, earlier administration of PTCy on days +1/+2 was associated with a less robust decrease in alloreactive CD4^+^ conventional T-cell proliferation at day +7 and later dosing on days +5/+6 was associated with a blunted relative recovery of T_regs_ by day +21, likely contributing in either case to the lower efficacy seen when compared with PTCy on days +3/+4 (6). Clinical success of PTCy was not achieved until the dose and timing were better optimized; in fact, early clinical studies using serial administration for 100 days of low dose PTCy suggested partial efficacy that was inferior to cyclosporine when each was combined with methylprednisolone (63). Moreover, it is possible that other chemotherapeutics may be more effective when given in different dosing schedules, and such optimal dosing schedules might vary depending on the relative metabolism of each drug. For example, MTX’s current dosing schedule for acute GVHD prophylaxis, which was derived from animal studies (45, 64, 65) and then tested clinically (46, 47, 66), is distinct from PTCy with administration on days +1, +3, +6, +/- +11. Nevertheless, additional studies on optimal timing for administration of other chemotherapeutics would be necessary to determine if the day +3/+4 timing is universally optimal or whether another chemotherapeutic administered in a different schedule or in combination with PTCy may produce similar or superior results to PTCy alone.

Our data showed no clear association between drug metabolism or resistance pathways and effective GVHD control. We had specifically chosen chemotherapeutics with varying involvement of ABC transporters and ALDH in resistance pathways (**Table 1**) to explore their relative efficacy. Interestingly, resistance patterns of the partially effective drugs (CY, MTX, and ARA-C) diverge (8, 12–14, 20–23), but may explain why each had differing effects on T-cell subset activation, proliferation, differentiation, and recovery kinetics. Yet, resistance patterns are not very dissimilar for the ineffective drugs compared with the effective drugs (**Table 1**) (8, 12–14, 19–36), suggesting either a small therapeutic index to effectively prevent GVHD or that the cellular impact of these drugs and/or their metabolism may be more complicated than we currently appreciate. Furthermore, MTX still facilitated the preferential recovery of T_regs_ by day +21 and even expanded T_regs_ at day +7. However, when considering ALDH versus ABC transporter activity, ABC transporters, which T_regs_ lack (67), likely play a comparatively larger role in resistance to MTX (**Table 1**) (12–14, 21–23, 30, 31, 36). By contrast, MTX has not been reported to be metabolized *via* ALDH which appears important for mediating T_reg_ resistance to cyclophosphamide (3, 4). Even so, the recovery kinetics of T_regs_ in MTX-treated mice were distinct from all other chemotherapeutics as was the balance of CD4^+^ versus CD8^+^ T cells. MTX uniquely facilitated increased percentages of T_regs_ at day +7, which may have mitigated effects of the increased percentages of activated (CD25^+^) alloreactive CD4^+^ conventional T cells at that timepoint. The divergence in some immunologic effects of MTX and PTCy suggests that GVHD control by MTX may occur *via* a different mechanism than PTCy and may explain why MTX is only effective for acute GVHD clinically, while PTCy can prevent both acute and chronic GVHD.

An additional interesting finding of this study is that BEN was ineffective in ameliorating severe and fatal GVHD in our MHC-haploidentical HCT model. Recently, post-transplantation BEN has shown promise in pre-clinical murine HCT studies (16) and mixed results in early phase clinical trials (17, 18). It is possible that our conflicting findings may be explained in part due to differences in models or relative doses of CY or BEN, which were higher in the CB6F1→CAF1/J model (16) than we have determined to be optimal at preventing GVHD in our B6C3F1→B6D2F1 model (5). Additionally, a recent clinical study examining post-transplantation BEN showed that patients developed severe cytokine release syndrome, often manifesting with liver dysfunction, at unacceptably high rates (18). This may parallel the ascites secondary to liver failure that developed in our BEN-treated mice. Ultimately, our results and the recent clinical study (18) suggest the need for caution in considering BEN as a suitable, safe, and effective alternative to PTCy until more mature clinical data are available.

In conclusion, our results provide further insight into the mechanisms of PTCy and the biology of GVHD prevention. The clinical and immunologic effects of cyclophosphamide given in the early post-transplant period appear unique and not fully reproducible by another alkylating agent or four other chemotherapeutics of multiple classes given over the same dosing schedule. Our data show that effective GVHD prophylaxis is associated with distinctive effects on constraining alloreactive conventional T-cell numerical reconstitution and facilitating preferential T_reg_ recovery at day +21, but also uncover that PTCy uniquely restrains alloreactive CD4^+^ conventional T-cell proliferation and differentiation. To what extent these findings hold true in patients, particularly those receiving adjunct immunosuppression beyond PTCy or to patients undergoing combined HCT/solid organ transplantation (68, 69), requires further exploration.

## Data Availability Statement

The raw data supporting the conclusions of this article will be made available by the authors, without undue reservation.

## Ethics Statement

The animal study was reviewed and approved by National Cancer Institute Animal Care and Use Committee.

## Author Contributions

CK designed the study. AH contributed to the study design. AH, NN, SK, RF, and AP performed the experiments. AH and CK analyzed the data. ME performed blinded assessments of histopathology. DV designed and performed the statistical analyses. All authors interpreted the data. AH and CK designed and created the tables and figures. AH and CK wrote the manuscript, and all authors revised the manuscript. All authors contributed to the article and approved the submitted version.

## Funding

This work was supported by the Intramural Research Program of the National Cancer Institute, National Institutes of Health (NIH), and the Lasker Foundation. This work was made possible through the NIH Medical Research Scholars Program, a public-private partnership supported jointly by the NIH and contributions to the Foundation for the NIH from the Doris Duke Foundation, Genentech, the American Association for Dental Research, and the Colgate-Palmolive Company. None of these supporting institutions or companies were involved in the study design, data collection, data analysis, data interpretation, manuscript preparation, or the decision to submit the manuscript for publication.

## Conflict of Interest

The authors declare that the research was conducted in the absence of any commercial or financial relationships that could be construed as a potential conflict of interest.

## Publisher’s Note

All claims expressed in this article are solely those of the authors and do not necessarily represent those of their affiliated organizations, or those of the publisher, the editors and the reviewers. Any product that may be evaluated in this article, or claim that may be made by its manufacturer, is not guaranteed or endorsed by the publisher.
