# Supplementary material for: Post-Transplantation Cyclophosphamide Uniquely Restrains Alloreactive CD4+ T-Cell Proliferation and Differentiation After Murine MHC-Haploidentical Hematopoietic Cell Transplantation
Source: Front Immunol. 2022 Feb 15;13:796349. doi: 10.3389/fimmu.2022.796349 (PMC8886236; doi:10.3389/fimmu.2022.796349)
Supplement: Supplementary file 1 [file DataSheet_1.pdf]

Supplementary Data for

Post-Transplantation Cyclophosphamide Uniquely Restrains Alloreactive CD4$^+$ T-cell Proliferation and Differentiation After Murine MHC-Haploidentical Hematopoietic Cell Transplantation

Ashley D. Hadjis,[1] Natalia S. Nunes,[1] Shanzay M. Khan,[1] Rochelle E. Fletcher,[1] Alessandra de Paula Pohl,[1] David J. Venzon,[2] Michael A. Eckhaus,[3] Christopher G. Kanakry[1]

[1]Experimental Transplantation and Immunotherapy Branch, Center for Cancer Research, National Cancer Institute, National Institutes of Health, Bethesda, Maryland, USA;

[2]Biostatistics and Data Management Section, Office of the Clinical Director, Center for Cancer Research, National Cancer Institute, National Institutes of Health, Bethesda, Maryland, USA;

[3]Division of Veterinary Resources, Office of Research Services, National Institutes of Health, Bethesda, Maryland, USA.

This PDF file includes:

Supplementary Table 1. Representative examples of histopathologic assessments at day +7.
Supplementary Table 2. Representative examples of histopathologic assessments at day +21.
Supplementary Figure 1. Methotrexate 0.1 mg/kg/day or 0.5 mg/kg/day is not superior to 1 mg/kg/day.
Supplementary Figure 2. Individual organ graft-versus-host disease histopathologic scores.
Supplementary Figure 3. Methotrexate and cyclophosphamide have opposite effects on the balance of CD4$^+$ versus CD8$^+$ T cells, distinct from all other chemotherapeutics.
Supplementary Figure 4. CD25 expression does not correlate with phosphorylation of STAT5 at day +7 in CD4$^+$Foxp3$^-$ conventional T cells.
Supplementary Figure 5. The effects of chemotherapeutics on alloreactive Vβ6$^+$ T-cell numbers mirror the broader effects seen on CD4$^+$ and CD8$^+$ T-cell subsets.
Supplementary Figure 6. Cyclophosphamide uniquely restrains T-cell differentiation at both day +7 and day +21.

Supplementary Table 1. Representative examples of histopathologic assessments at day +7 (page 1 of 2).

	Skin	Liver	Stomach	Small Intestine (SI)	Cecum	Colon
**TCD BM, Splen, Vehicle D +3, +4**<br>Total Score: 12<br>Skin: 1 SI: 3<br>Liver: 2 Cecum: 2<br>Stomach: 2 Colon: 2	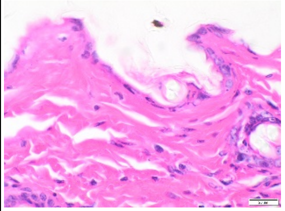	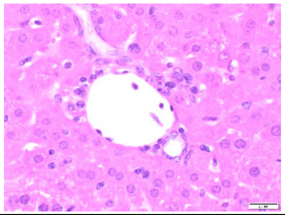	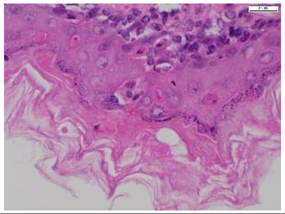	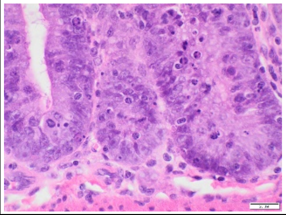	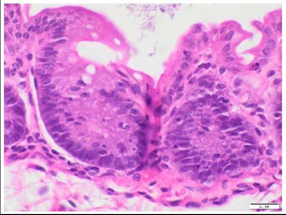	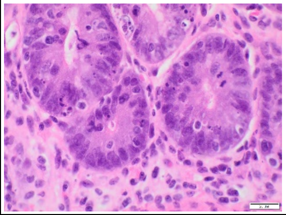
**TCD BM, Splen, CY 25 mg/kg D +3, +4**<br>Total Score: 7<br>Skin: 0 SI: 2<br>Liver: 1 Cecum: 2<br>Stomach: 1 Colon:1	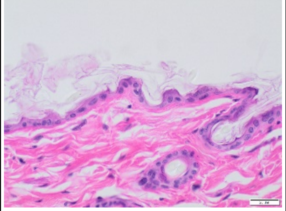	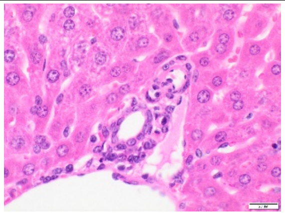	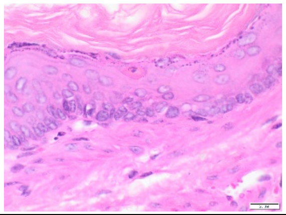	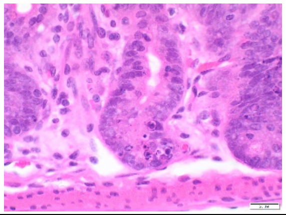	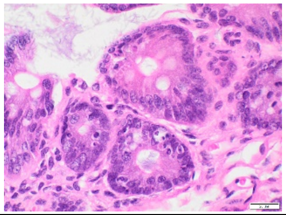	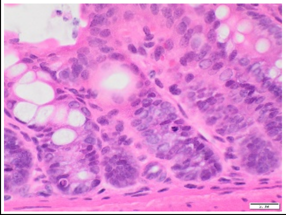
**TCD BM, Splen, MTX 5 mg/kg D +3, +4**<br>Total Score: 7<br>Skin: 0 SI: 2<br>Liver: 1 Cecum: 1<br>Stomach: 1 Colon: 2	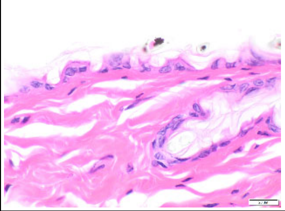	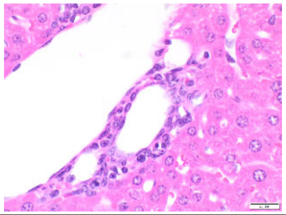	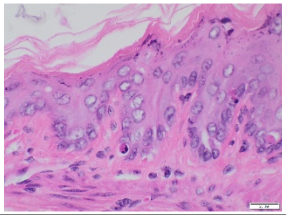	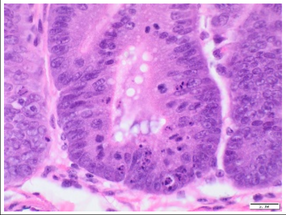	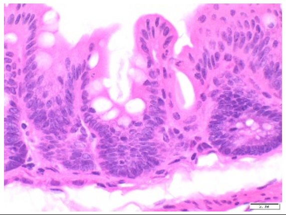	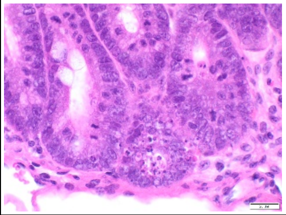
**TCD BM, Splen, BEN 10 mg/kg D +3, +4**<br>Total Score: 10<br>Skin: 1 SI: 3<br>Liver: 1 Cecum: 1<br>Stomach: 2 Colon: 2	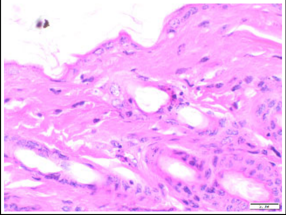	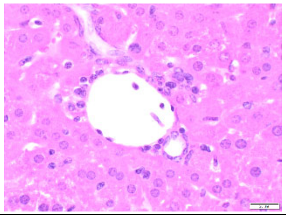	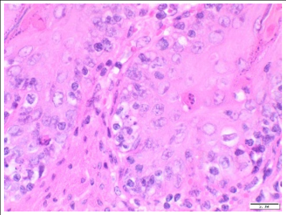	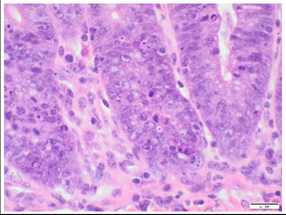	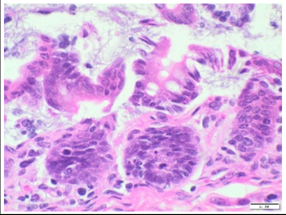	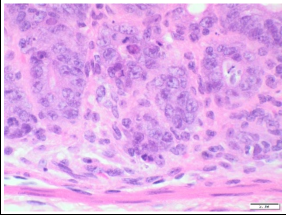
**TCD BM, Splen, PTX 1 mg/kg D +3, +4**<br>Total Score: 10<br>Skin: 0 SI: 2<br>Liver: 2 Cecum: 2<br>Stomach: 2 Colon: 2	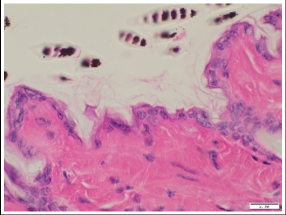	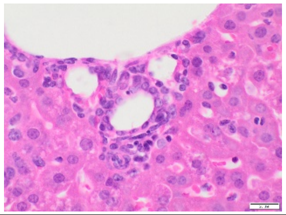	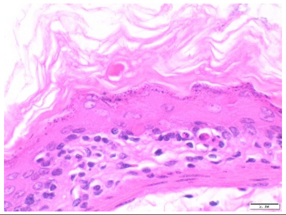	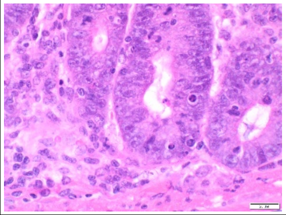	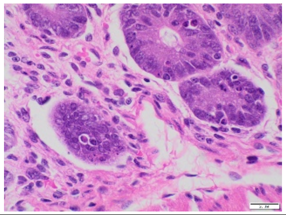	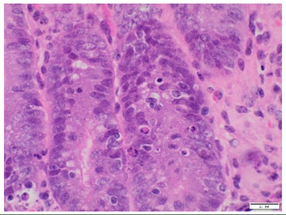

Supplementary Table 1. Representative examples of histopathologic assessments at day +7 (page 2 of 2).

	Skin	Liver	Stomach	Small Intestine (SI)	Cecum	Colon
**TCD BM, Splen, VCR 0.05 mg/kg D +3, +4**<br>Total Score: 10<br>Skin: 1 SI: 3<br>Liver: 1 Cecum: 1<br>Stomach: 2 Colon: 2	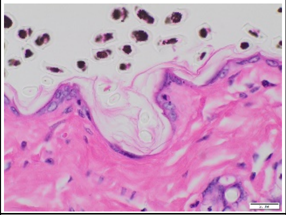	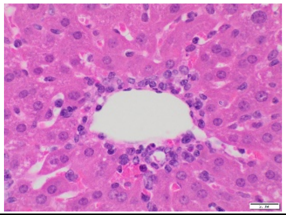	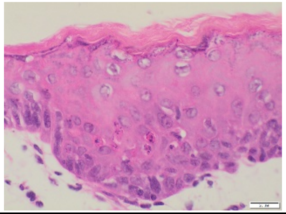	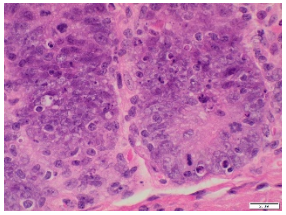	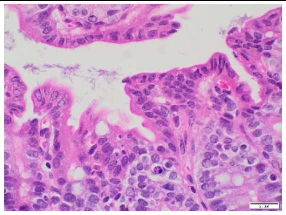	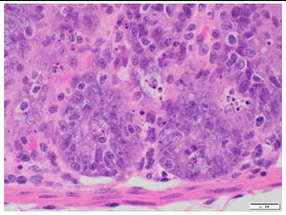
**TCD BM, Splen, ARA-C 25 mg/kg D +3, +4**<br>Total Score: 10<br>Skin: 0 SI: 3<br>Liver: 2 Cecum: 2<br>Stomach: 1 Colon: 2	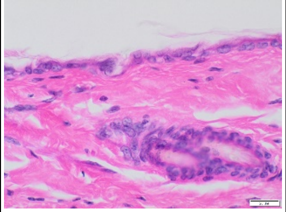	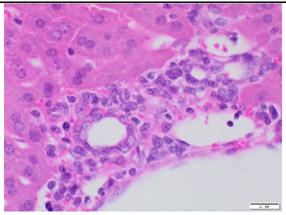	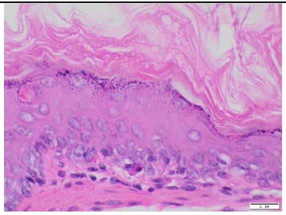	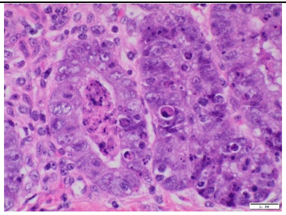	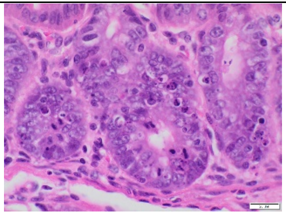	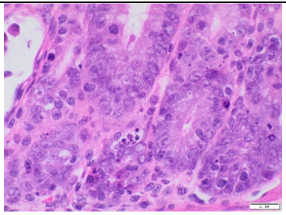

Notes: D, day; TCD, T-cell depleted; BM, bone marrow; Splen, splenocytes; CY, cyclophosphamide; MTX, methotrexate; BEN, bendamustine; PTX, paclitaxel; VCR, vincristine; ARA-C, cytarabine.

Supplementary Table 2. Representative examples of histopathologic assessments at day +21 (page 1 of 2).

	Skin	Liver	Stomach	Small Intestine (SI)	Cecum	Colon
**TCD BM, Splen, Vehicle D +3, +4**<br>Total Score: 11<br>Skin: 2 SI: 2<br>Liver: 1 Cecum: 2<br>Stomach: 2 Colon: 2	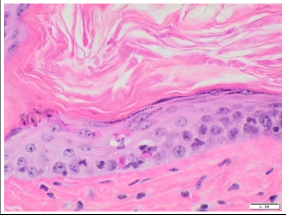	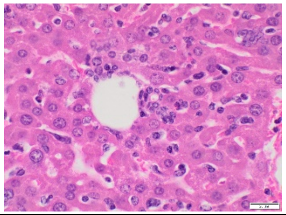	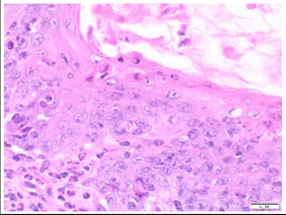	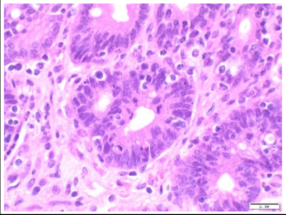	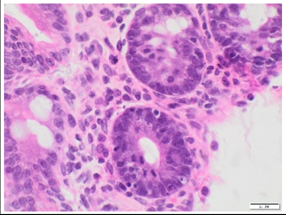	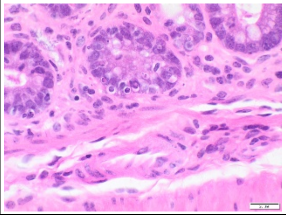
**TCD BM, Splen, CY 25 mg/kg D +3, +4**<br>Total Score: 5<br>Skin: 1 SI: 1<br>Liver: 1 Cecum: 1<br>Stomach: 0 Colon:1	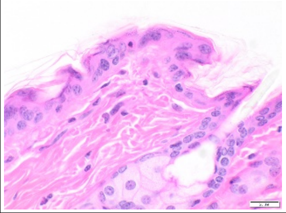	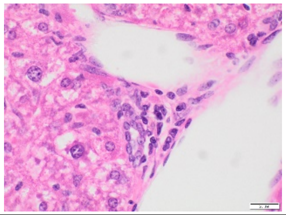	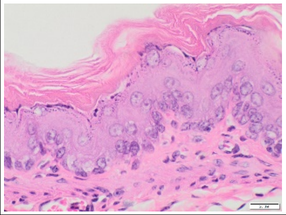	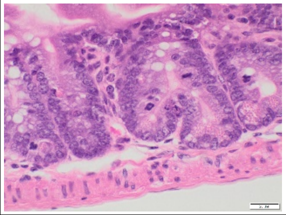	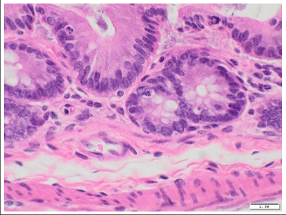	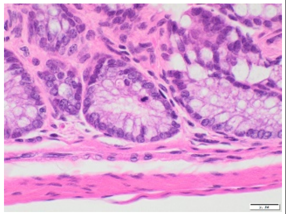
**TCD BM, Splen, MTX 5 mg/kg D +3, +4**<br>Total Score: 7<br>Skin: 1 SI: 1<br>Liver: 2 Cecum: 1<br>Stomach: 1 Colon: 1	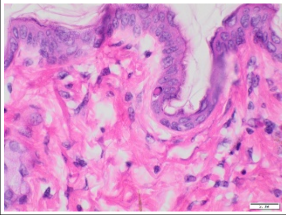	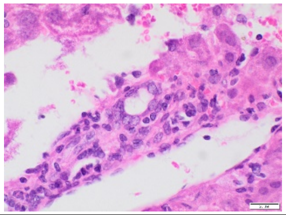	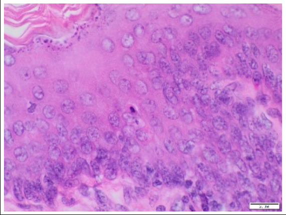	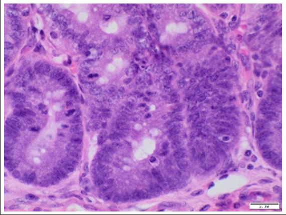	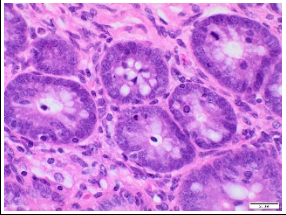	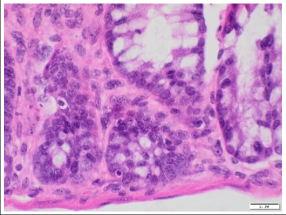
**TCD BM, Splen, BEN 10 mg/kg D +3, +4**<br>Total Score: 12<br>Skin: 2 SI: 2<br>Liver: 3 Cecum: 1<br>Stomach: 2 Colon: 2	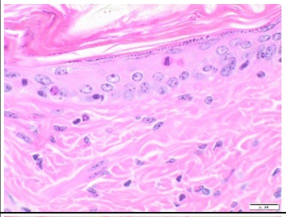	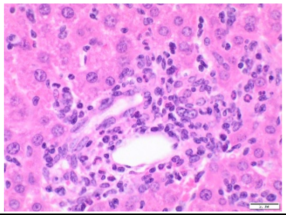	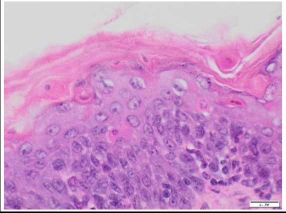	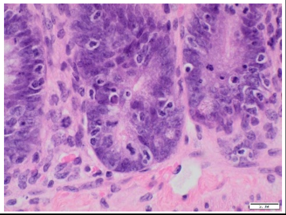	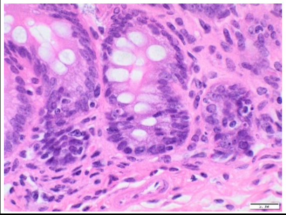	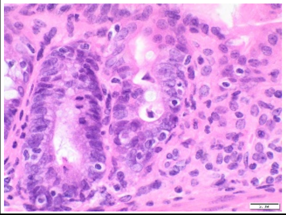
**TCD BM, Splen, PTX 1 mg/kg D +3, +4**<br>Total Score: 13<br>Skin: 2 SI: 3<br>Liver: 2 Cecum: 2<br>Stomach: 2 Colon: 2	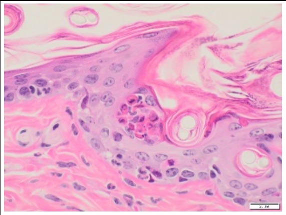	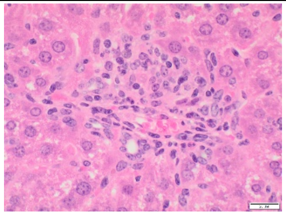	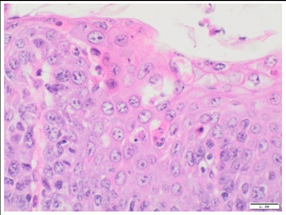	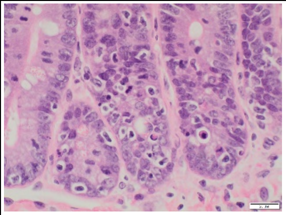	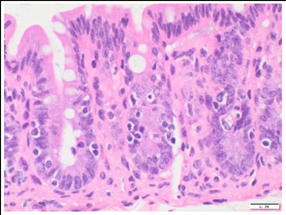	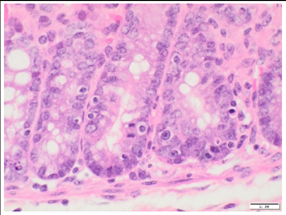

Supplementary Table S2. Representative examples of histopathologic assessments at day +21 (page 2 of 2).

	Skin	Liver	Stomach	Small Intestine (SI)	Cecum	Colon
**TCD BM, Splen, VCR 0.05 mg/kg D +3, +4**<br>Total Score: 13<br>Skin: 2 SI: 2<br>Liver: 2 Cecum: 2<br>Stomach: 3 Colon: 2	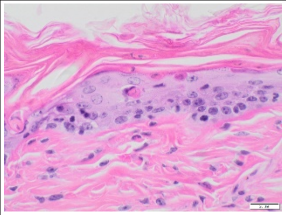	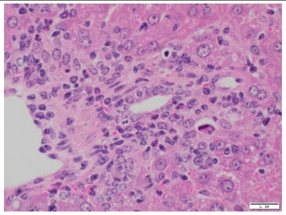	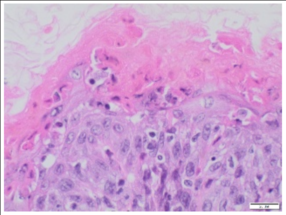	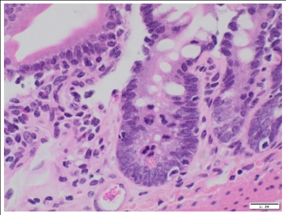	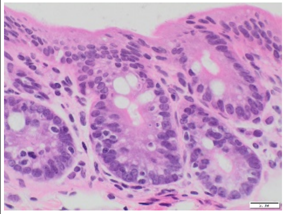	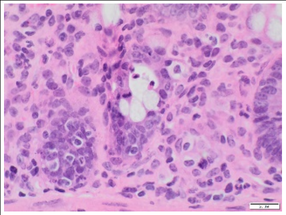
**TCD BM, Splen, ARA-C 25 mg/kg D +3, +4**<br>Total Score: 7<br>Skin: 1 SI: 2<br>Liver: 1 Cecum: 1<br>Stomach: 1 Colon: 1	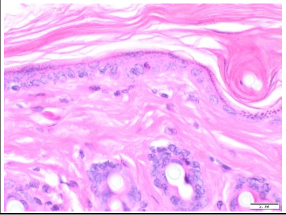	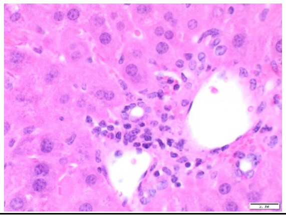	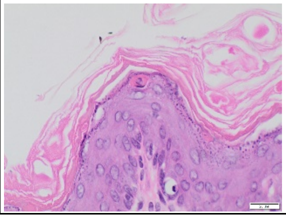	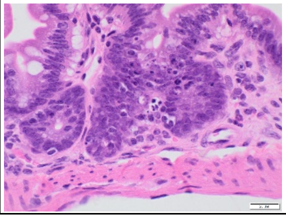	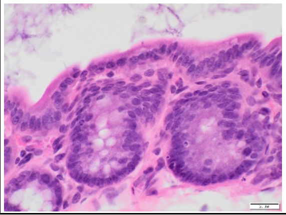	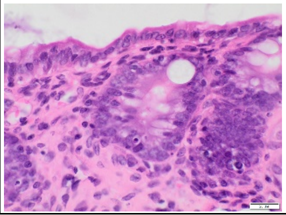

Notes: D, day; TCD, T-cell depleted; BM, bone marrow; Splen, splenocytes; CY, cyclophosphamide; MTX, methotrexate; BEN, bendamustine; PTX, paclitaxel; VCR, vincristine; ARA-C, cytarabine.

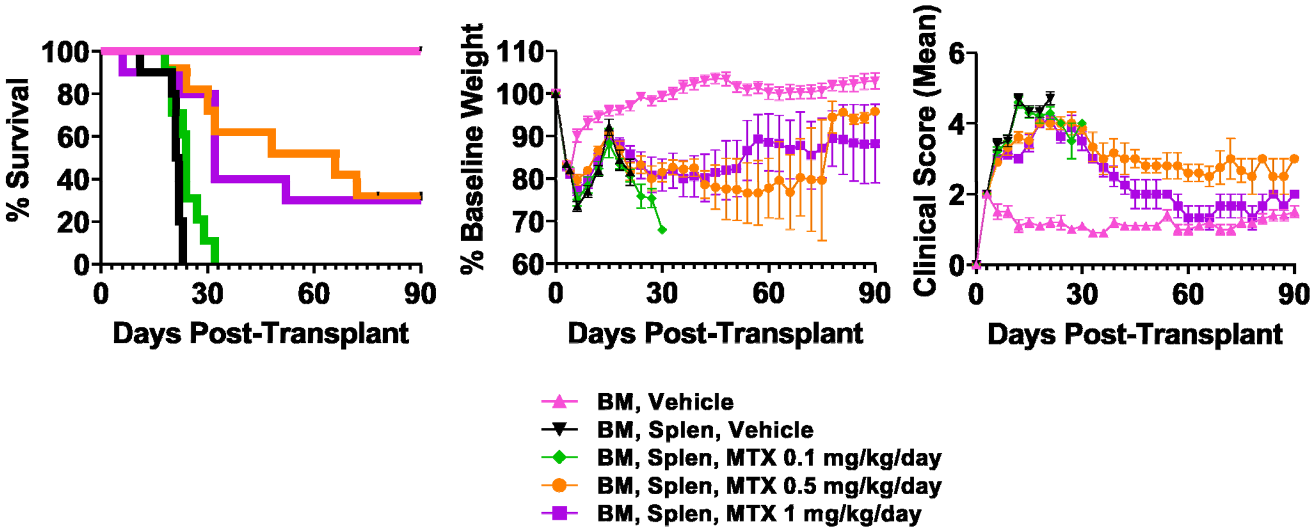

Supplementary Figure 1. Methotrexate 0.1 mg/kg/day or 0.5 mg/kg/day is not superior to 1 mg/kg/day. On day 0, recipient 10-12-week-old female B6D2F1 mice were irradiated to 10.5 Gy in a single fraction and transplanted 6-8 hours later via intravenous injection with 10 x 10^6 T-cell-depleted bone marrow (BM) cells +/- 40 x 10^6 red-blood-cell-depleted splenocytes (Splen) from 10-12-week-old female B6C3F1 donors. Phosphate buffered saline (PBS) vehicle or methotrexate (MTX) was administered intraperitoneally on days +3 and +4. Very low doses of methotrexate were tested compared with the lowest dose (1 mg/kg/day) tested in the experiments shown in **Figure 1** to ensure that MTX 5 mg/kg/day was the optimal dose. Combined results from two independent experiments are shown with n=5/group/experiment.

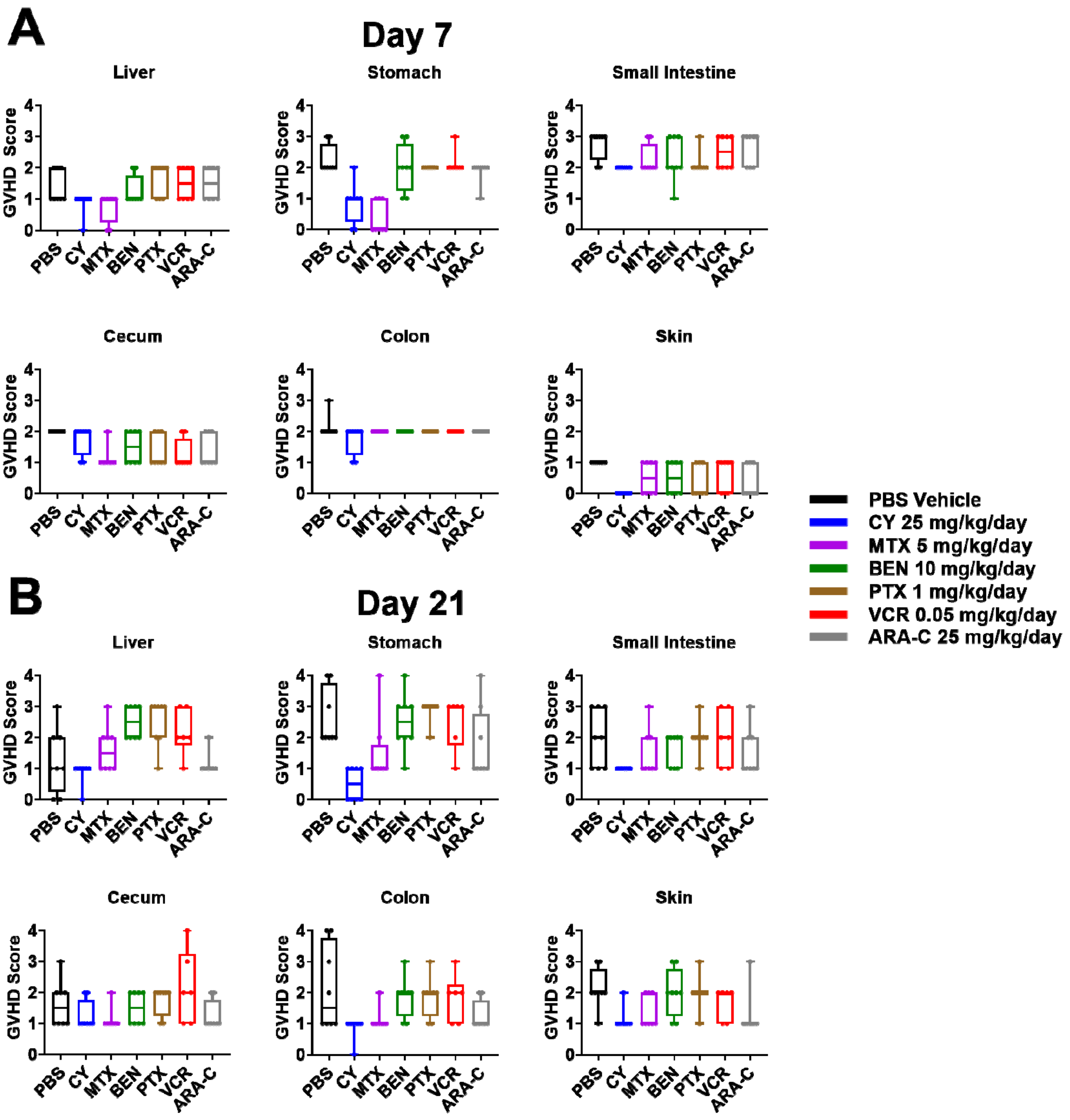

Supplementary Figure 2. Individual organ graft-versus-host disease histopathologic scores. Mice were transplanted as in **Supplementary Figure 1**. PBS vehicle or the chemotherapeutic of interest was administered intraperitoneally on days +3 and +4. Mice were euthanized and assessed for histopathology of GVHD target organs at **(A)** day +7 or **(B)** day +21. Individual organ results shown here were summed to generate the total GVHD histopathologic scores shown in **Figure 2B**. Combined results from two independent experiments are shown with n=4/group/experiment except for VCR at day +21 (n=6 total) due to excess early deaths in one experiment prior to day +21.

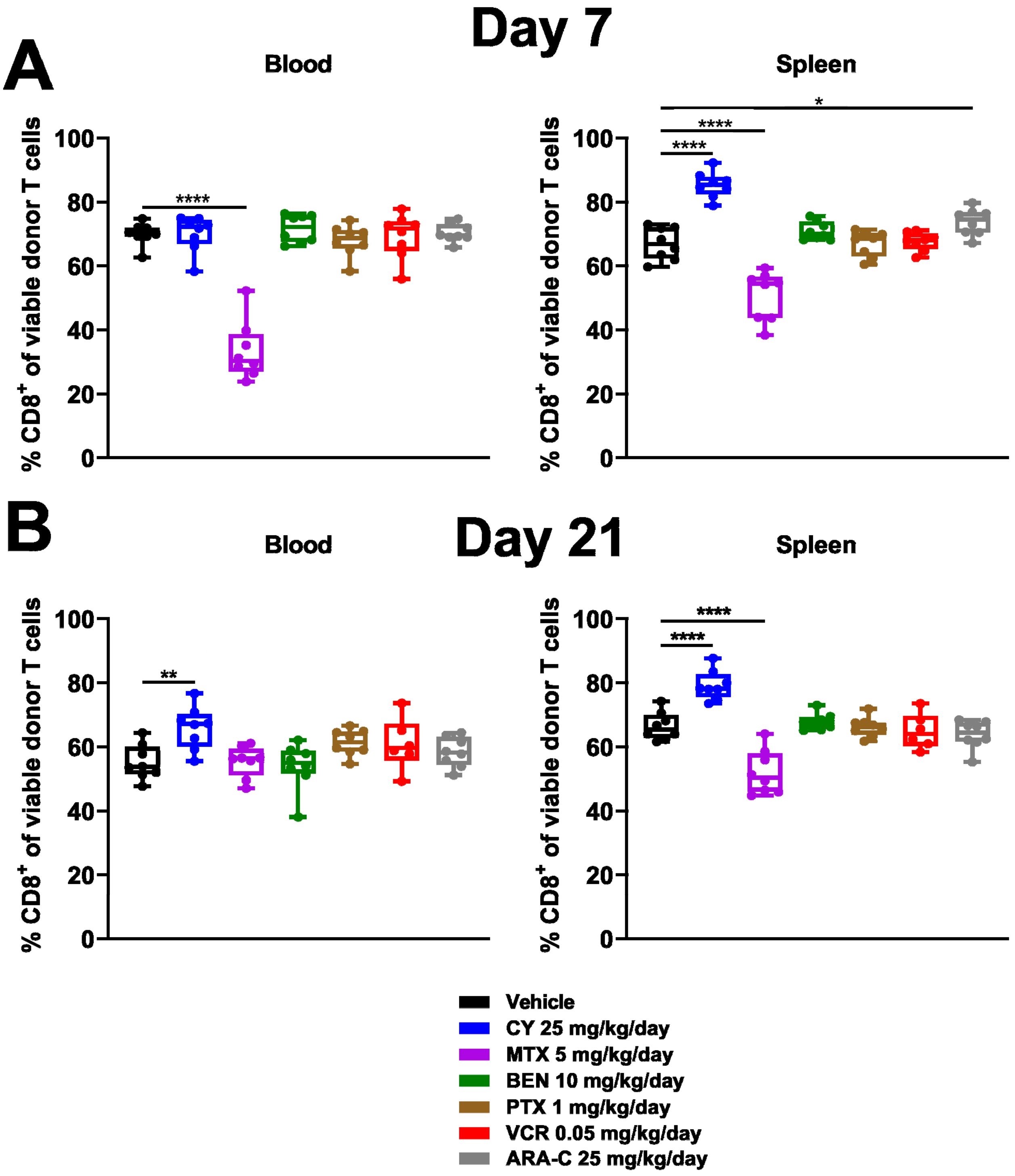

Supplementary Figure 3. Methotrexate and cyclophosphamide have opposite effects on the balance of $CD4^+$ versus $CD8^+$ T cells, distinct from all other chemotherapeutics. Data in **Figure 3C-D** are included here but instead show the percentage of T cells that were $CD8^+CD4^-$ at **(A)** day +7 and **(B)** day +21. Combined results from two independent experiments are shown with n=4/group/experiment except for VCR at day +21 (n=6 total) due to excess early deaths in one experiment prior to day +21. $^*p<0.05$, $^{**}p<0.01$, and $^{****}p<0.0001$ on one-way ANOVA followed by the Holm-Sidak post hoc test using the vehicle-treated group as the control. Only significant results are shown; all other comparisons between treatment groups and the vehicle group are non-significant.

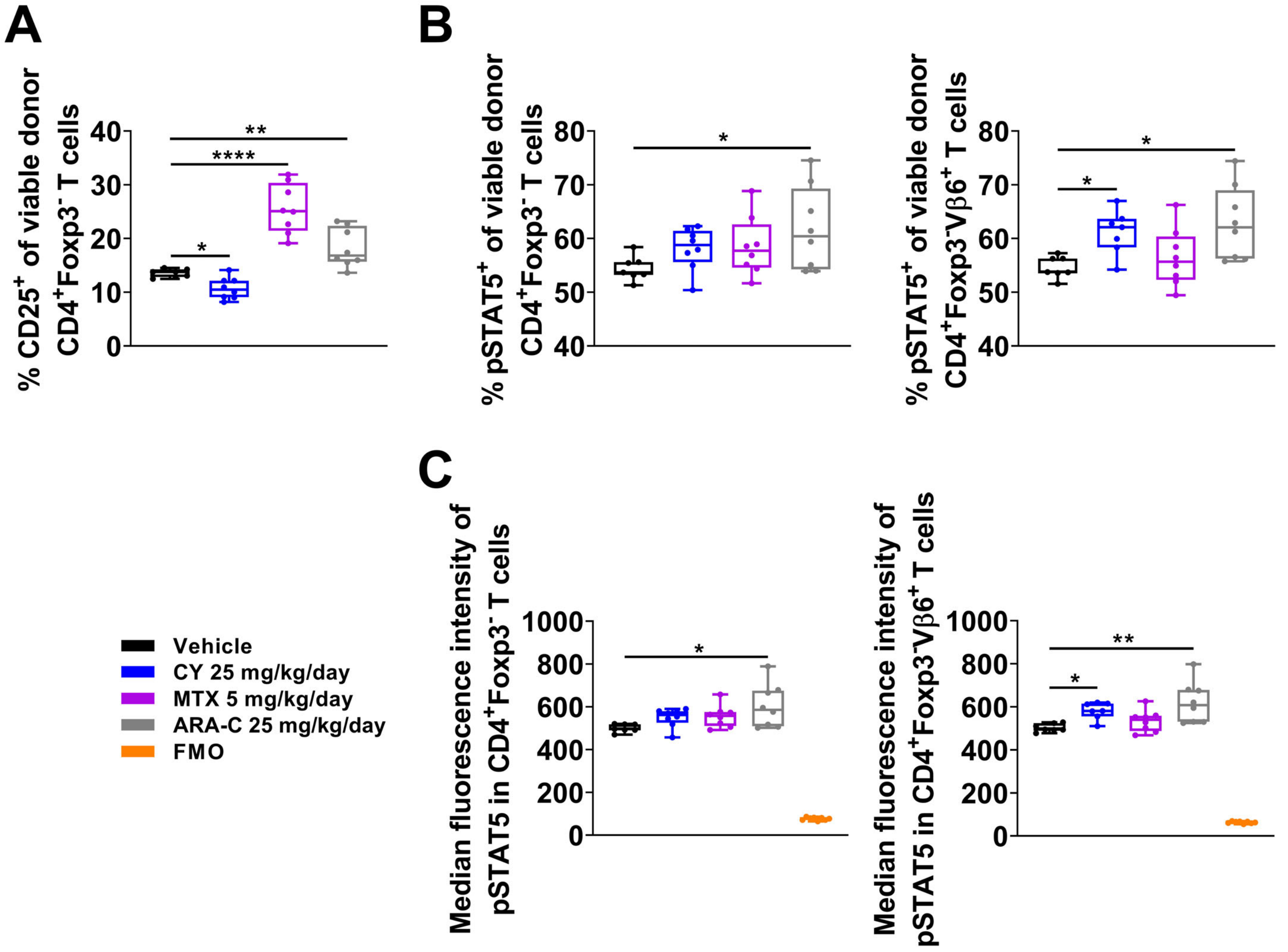

Supplementary Figure 4. CD25 expression does not correlate with phosphorylation of STAT5 at day +7 in CD4$^+$Foxp3$^-$ conventional T cells. Phosphorylation of STAT5 (pSTAT5), a transcription factor that is involved in T-cell activation including subsequent expression of CD25, was assessed at day +7 within spleens to further characterize the impact of the partially effective chemotherapeutics (CY, MTX, and ARA-C). **(A)** Reproducing the results shown in **Figure 4B-C**, CY resulted in decreased percentages of CD4$^+$Foxp3$^-$ conventional T cells that were CD25$^+$, while MTX and ARA-C led to higher percentages. **(B-C)** Yet, CY-treated mice did not have lower **(B)** percentages of pSTAT5$^+$ cells or **(C)** median fluorescence intensity (MFI) of pSTAT5 expression in either all CD4$^+$Foxp3$^-$ conventional T cells or alloreactive (Vβ6$^+$) CD4$^+$Foxp3$^-$ conventional T cells. Fluorescence-minus-one (FMO) controls (one for each treatment group per experiment) are shown for reference of background PE signal. Combined results from two independent experiments are shown with n=3-4/group/experiment. *$p<0.05$, **$p<0.01$, and ****$p<0.0001$ on one-way ANOVA followed by the Holm-Sidak post hoc test using the vehicle-treated group as the control. Only significant results are shown; all other comparisons between treatment groups and the vehicle group are non-significant.

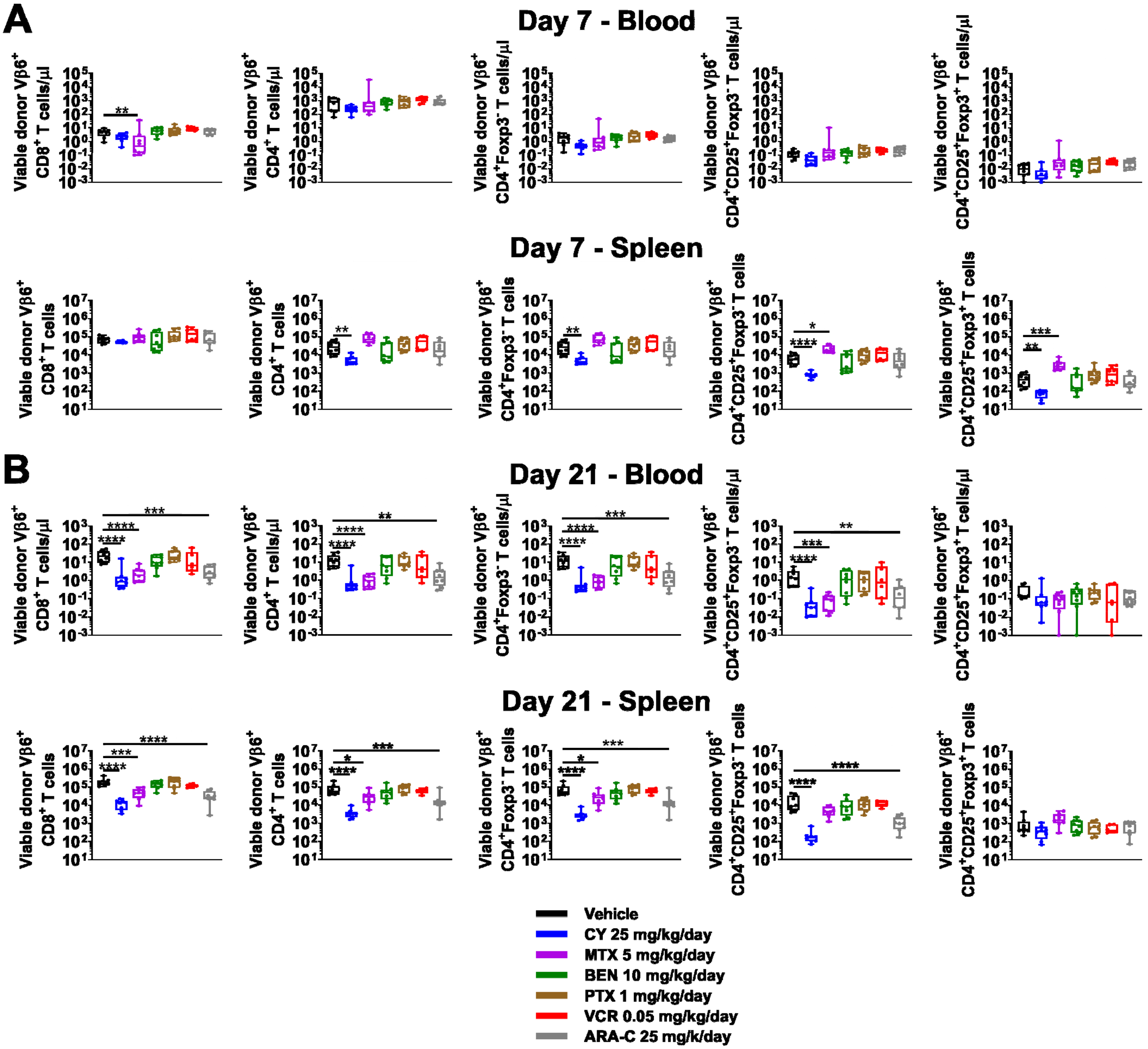

Supplementary Figure 5. The effects of chemotherapeutics on alloreactive Vβ6+ T-cell numbers mirror the broader effects seen on CD4+ and CD8+ T-cell subsets. Numerical count recovery corresponding to percentage data in **Figure 5** are shown. **(A)** Alloreactive Vβ6+ T cells were not eliminated by any chemotherapeutic at day +7. In fact, total numbers of alloreactive Vβ6+ T cells generally persisted in the same log range across all treatment groups, except CY reduced total numbers of Vβ6+ CD4+ T-cell subsets as it did for overall CD4+ T cells shown in **Figure 3A**. MTX-treated mice had higher total numbers of Vβ6+ CD4+ T cells, particularly CD4+CD25+Foxp3- T cells and CD4+CD25+Foxp3+ T_{regs}, again similar to the effects broadly on CD4+ T cells. **(B)** At day +21, the three partially effective chemotherapeutics (CY, MTX, ARA-C) all constrained recovery of Vβ6+ T cells similar to their effects on conventional T cells more broadly as shown in **Figure 3B**. Combined results from two independent experiments are shown with n=4/group/experiment except for VCR at day +21 (n=6 total) due to excess early deaths in one experiment prior to day +21. *p<0.05, **p<0.01, ***p<0.001, and ****p<0.0001 on one-way ANOVA followed by the Holm-Sidak post hoc test using the vehicle-treated group as the control. Only significant results are shown; all other comparisons between treatment groups and the vehicle group are non-significant.

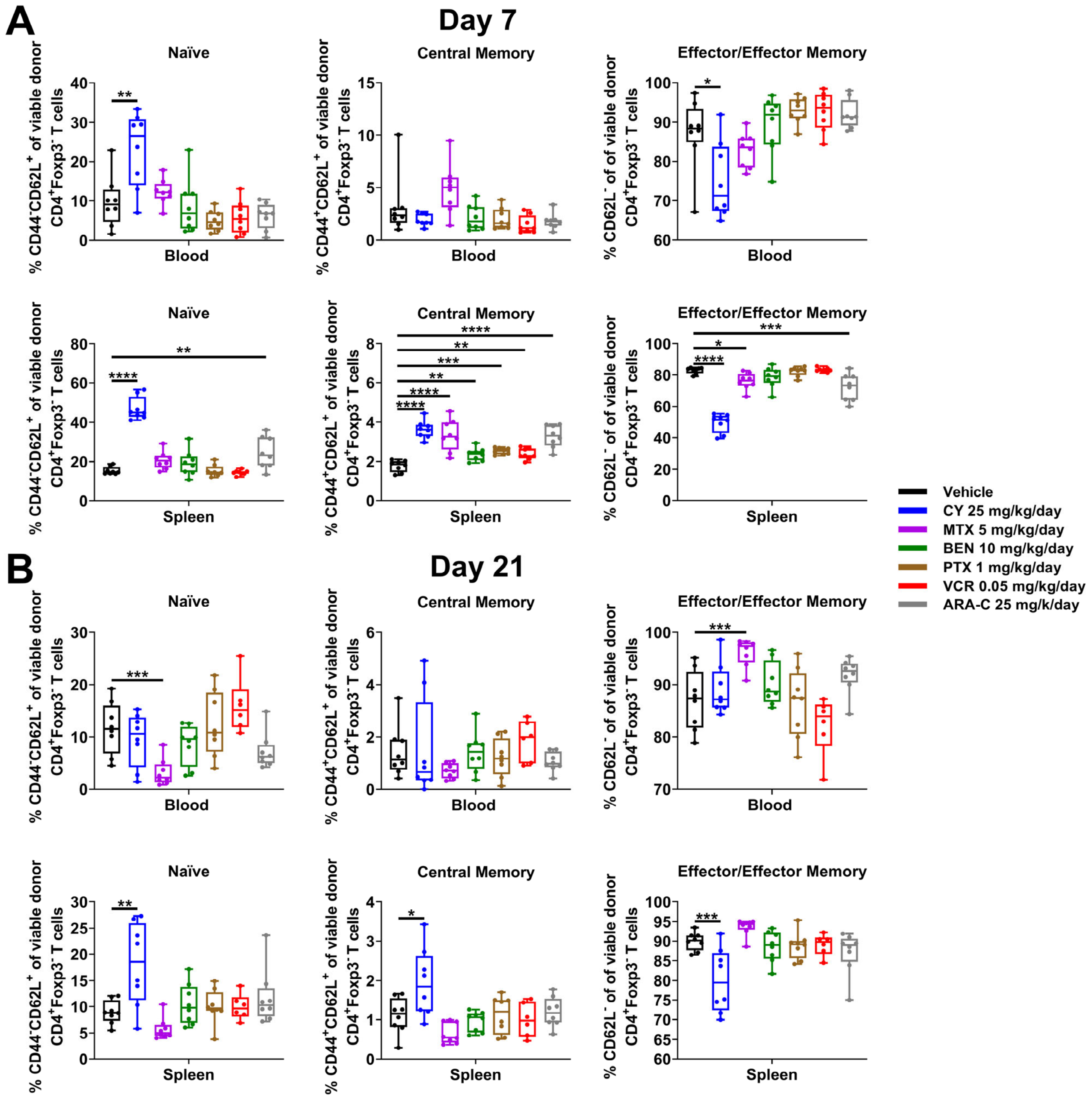

Supplementary Figure 6. Cyclophosphamide uniquely restrains T-cell differentiation at both day +7 and day +21. Shown are the effects on unselected CD4$^+$Foxp3$^-$ T cells corresponding to the CD4$^+$Foxp3$^-$Vβ6$^+$ data shown in **Figure 7**. **(A)** At day +7, CY decreased percentage of CD4$^+$Foxp3$^-$ T cells that were phenotypically effector/effector memory (CD62L$^-$). Therefore, percentages of naïve (CD44$^-$CD62L$^+$) and central memory (CD44$^+$CD62L$^+$) CD4$^+$Foxp3$^-$ T cells were increased by CY at day +7. To a lesser extent, this same effect also was seen in MTX- and ARA-C-treated mice. **(B)** This effect generally persisted at day +21 in CY-treated mice, but it was lost in both MTX- and ARA-C-treated mice. In fact, MTX had more highly differentiated cells at day +21. Combined results from two independent experiments are shown with n=4/group/experiment except for VCR at day +21 (n=6 total) due to excess early deaths in one experiment prior to day +21. *p<0.05, **p<0.01, ***p<0.001, and ****p<0.0001 on one-way ANOVA followed by the Holm-Sidak post hoc test using the vehicle-treated group as the control. Only significant results are shown; all other comparisons between treatment groups and the vehicle group are non-significant.
